# Mechanism and endoscopic‐treatment‐induced evolution of biliary non‐anastomotic stricture after liver transplantation revealed by single‐cell RNA sequencing

**DOI:** 10.1002/ctm2.1622

**Published:** 2024-03-14

**Authors:** Zhaoyi Wu, Danqing Liu, Yanjiao Ou, Zeliang Xu, Gang Heng, Wei Liu, Nengsheng Fu, Jingyi Wang, Di Jiang, Lang Gan, Jiahong Dong, Xiaojun Wang, Zhiyu Chen, Leida Zhang, Chengcheng Zhang

**Affiliations:** ^1^ Department of Hepatobiliary Surgery Southwest Hospital, Third Military Medical University (Army Medical University) Chongqing China; ^2^ Department of General Surgery PLA Middle Military Command General Hospital Wuhan China; ^3^ Hepatopancereatobiliary Center, Beijing Tsinghua Changgung Hospital, School of Clinical Medicine Tsinghua University Beijing People's Republic of China

**Keywords:** atlas of bile duct microenvironment, endoscopic treatment, epithelial cell degeneration and regeneration, liver transplantation, non‐anastomotic stricture, scRNA‐seq

## Abstract

**Background:**

Biliary complications, especially non‐anastomotic stricture (NAS), are the main complications after liver transplantation. Insufficient sampling and no recognized animal models obstruct the investigation. Thus, the mechanisms and alterations that occur during endoscopic treatment (ET) of NAS remain unclear.

**Methods:**

Samples were obtained with endoscopic forceps from the hilar bile ducts of NAS patients receiving continuous biliary stent implantation after diagnosis. Retrospective analysis of multiple studies indicated that the duration of ET for NAS was approximately 1–2 years. Thus, we divided the patients into short‐term treatment (STT) and long‐term treatment (LTT) groups based on durations of less or more than 1 year. Samples were subjected to single‐cell RNA sequencing. Transcriptomic differences between STT and normal groups were defined as the NAS mechanism. Similarly, alterations from STT to LTT groups were regarded as endoscopic‐treatment‐induced evolution.

**Results:**

In NAS, inflammation and immune‐related pathways were upregulated in different cell types, with nonimmune cells showing hypoxia pathway upregulation and immune cells showing ATP metabolism pathway upregulation, indicating heterogeneity. We confirmed a reduction in bile acid metabolism‐related *SPP1^+^* epithelial cells in NAS. Increases in proinflammatory and profibrotic fibroblast subclusters indicated fibrotic progression in NAS. Furthermore, immune disorders in NAS were exacerbated by an increase in plasma cells and dysfunction of NK and NKT cells. ET downregulated multicellular immune and inflammatory responses and restored epithelial and endothelial cell proportions.

**Conclusions:**

This study reveals the pathophysiological and genetic mechanisms and evolution of NAS induced by ET, thereby providing preventive and therapeutic insights into NAS.

**Highlights:**

- For the first time, single‐cell transcriptome sequencing was performed on the bile ducts of patients with biliary complications.
- scRNA‐seq analysis revealed distinct changes in the proportion and phenotype of multiple cell types during Nonanastomotic stricture (NAS) and endoscopic treatment.
- A reduction in bile acid metabolism‐related SPP1+ epithelial cells and VEGFA+ endothelial cells, along with explosive infiltration of plasma cells and dysfunction of T and NK cells in NAS patients.
- SPP1+ macrophages and BST2+ T cells might serve as a surrogate marker for predicting endoscopic treatment.

## Graphical abstract

1. For the first time, single‐cell transcriptome sequencing was performed on the bile ducts of patients with biliary complications.
2. This study reveals the mechanism of NAS and the evolution of cells and genes during endoscopic treatment.
3. A reduction in bile acid metabolism‐related SPP1+ epithelial cells and VEGFA+ endothelial cells, along with explosive infiltration of plasma cells and dysfunction of T‐ and NK cells in NAS patients.

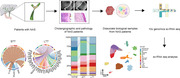

## 1 INTRODUCTION

Biliary complications (BCs), which have complex aetiologies and are difficult to treat, are the leading complications after liver transplantation (LT), occurring in approximately 20% of patients.^1^ The main type of BC, non‐anastomotic stricture (NAS) manifests as strictures or irregularities in the biliary duct beyond the surgical anastomosis and independently affects the survival of recipients.^2^, ^3^ NAS frequently involves intrahepatic and multifocal lesions of biliary trees. Risk factors for NAS are heterogeneous and complicated,^4^ and the current knowledge regarding NAS has been acquired only from clinical analysis or histological observation; thus, the molecular mechanism of NAS remains unknown.^5^, ^6^ Because it is difficult to obtain human NAS tissues and construct animal models, the aetiology of NAS has not been well illustrated. Continuous endoscopic biliary stent implantation by endoscopic retrograde cholangiopancreatography (ERCP) is the first‐line recommendation for NAS treatment with favourable effects.^4^ However, the cellular and genetic features of the NAS microenvironment and the evolution of the bile duct induced by endoscopic treatment (ET) are unclear.

The bile duct tissues of NAS patients undergo a gradual onset of epithelial injury due to chronic ischemia and hypoxia that manifests only after a significant delay, typically within the first 3 to 6 months following transplantation, but potentially even later.^7^ NAS is the consequence of degeneration and remodelling of biliary trees. All cells in the microenvironmental niche, including biliary epithelial cells, participate in this process. Persistent hypoxia, damage, and impaired regeneration of biliary epithelial cells play key roles in the pathogenesis of NAS.^5^, ^8^ The progenitors of biliary epithelial cells and the peribiliary glands are able to regenerate biliary epithelial cells, and injury to these glands is associated with NAS.^9^ In addition, insufficient vascular perfusion and injury to the vascular plexus of the bile duct niche predict NAS incidence.^9^, ^10^ Lymphocytes are associated with bile duct regeneration in transplant livers.^6^ Specific immune cells promote biliary epithelial cell proliferation in chronic liver diseases.^5^ Even though the pathology of NAS and its correlation with BCs have been reported previously, the cellular and genetic basis for NAS remains unknown. Tissue healing is the consequence of epithelial repair and microenvironmental remodeling,^11^ which are involved in ET of NAS. However, the healing of biliary trees has been assessed according to radiological and liver function test results only; thus, the mechanism is unknown.

Single‐cell RNA sequencing (scRNA‐seq) is one approach for dissecting the heterogeneity and interaction of cells in complex biological environments. By analyzing the transcriptomic alterations and evolution of cells, scRNA‐seq can create atlases illuminating the processes of diseases and treatments.^12^ However, scRNA‐seq has been rarely applied to investigate LT,^13^ and no scRNA‐seq study has been proposed to assess NAS. Unlike the acquisition of samples in other diseases, sampling of biliary trees in NAS patients by endoscopy is difficult, and the specimen size is quite small. Because scRNA‐seq enables transcriptomic analyses of individual cells with high throughput, it is a potentially valuable and powerful tool for NAS research.

Biliary stents need to be replaced every few months during the whole ET period.^4^ Therefore, samples for scRNA‐seq can be collected from the hilar bile ducts of NAS patients with endoscopic forceps during each ERCP procedure. As reported in many studies and based on observations at our centre, the ET duration is approximately 1−2 years with remission of stricture and normalization of liver function.^14^, ^15^, ^16^ In this study, we set 1 year as the cutoff value to divide NAS patients into short‐term treatment (STT) and long‐term treatment (LTT) groups. As it was difficult to acquire samples from NAS patients receiving first‐time ET that met the sequencing requirements due to the presence of excessive dead cells, the atlas of the NAS mechanism was elucidated by comparing STT samples to normal samples from normal bile ducts. The cellular and genetic evolution induced by ET was assessed according to the transcriptomic differences between STT and LTT samples. This transcriptomic atlas of NAS samples comprehensively resolved the changes in cells and genes with high throughput, ultimately illustrating the mechanism of NAS and the evolution of NAS induced by ET. To our knowledge, this is the first study revealing the cellular and genetic signatures of NAS by exploiting the strengths of scRNA‐seq. These findings reported here could provide new preventive and therapeutic insights into NAS.

## 2 MATERIALS AND METHODS

### 2.1 Tissue preparation

To elucidate the cellular composition and transcriptomic landscape of NAS and its evolution during ET, we performed scRNA‐seq on ten NAS patients receiving ERCP and ET at Southwest Hospital, Third Military Medical University (Army Medical University), Chongqing, China. Biopsy samples were acquired by either of the two following methods: (1) After biliary cannulation, anatomical features and biliary strictures were observed on the cholangiogram, biopsy forceps (JRQ‐Y2323‐PAC, Fuyang Jingrui Medical Technology Co, Ltd.) were inserted into the bile duct through the duodenal papilla under fluoroscopy, to the stricture sites in the hilar bile duct; the forceps were opened and pressed against the bile duct. A total of 3−6 tissue specimens were obtained by forceps sampling. (2) The SpyGlass system (Boston Scientific) was placed through the duodenal papilla into the bile duct. Under direct visualization, the bile duct was closely examined, and the SpyBite (Boston Scientific) instrument was carefully inserted into the planned biopsy site (hilum). Each planned biopsy site was sampled using the SpyBite tool under direct visualization to obtain 3−6 tissue specimens. We utilized scissors to scrape the mucosal and middle layers of bile duct tissue from two donor sources as the normal group.

### 2.2 Rat

All specific pathogen‐free male Sprague–Dawley rats, aged 6−8 weeks and weighing 200−220 g, were bought from Chongqing Ensiweier Co. All experiments were performed in accordance with guidelines of Institutional Animal Care and Use Committee of Army Medical University (AMUWEC20224101). We divided the extrahepatic bile duct of rats into two groups: the extrahepatic hilar bile duct and the common bile duct.

### 2.3 Single‐cell suspension preparation for scRNA‐seq and verification experiments

The details are presented in the Supporting Information Methods (Table [Link], [Link], [Link]).

## 3 RESULTS

### 3.1 Characteristics of NAS patients with ET

**FIGURE 1:**
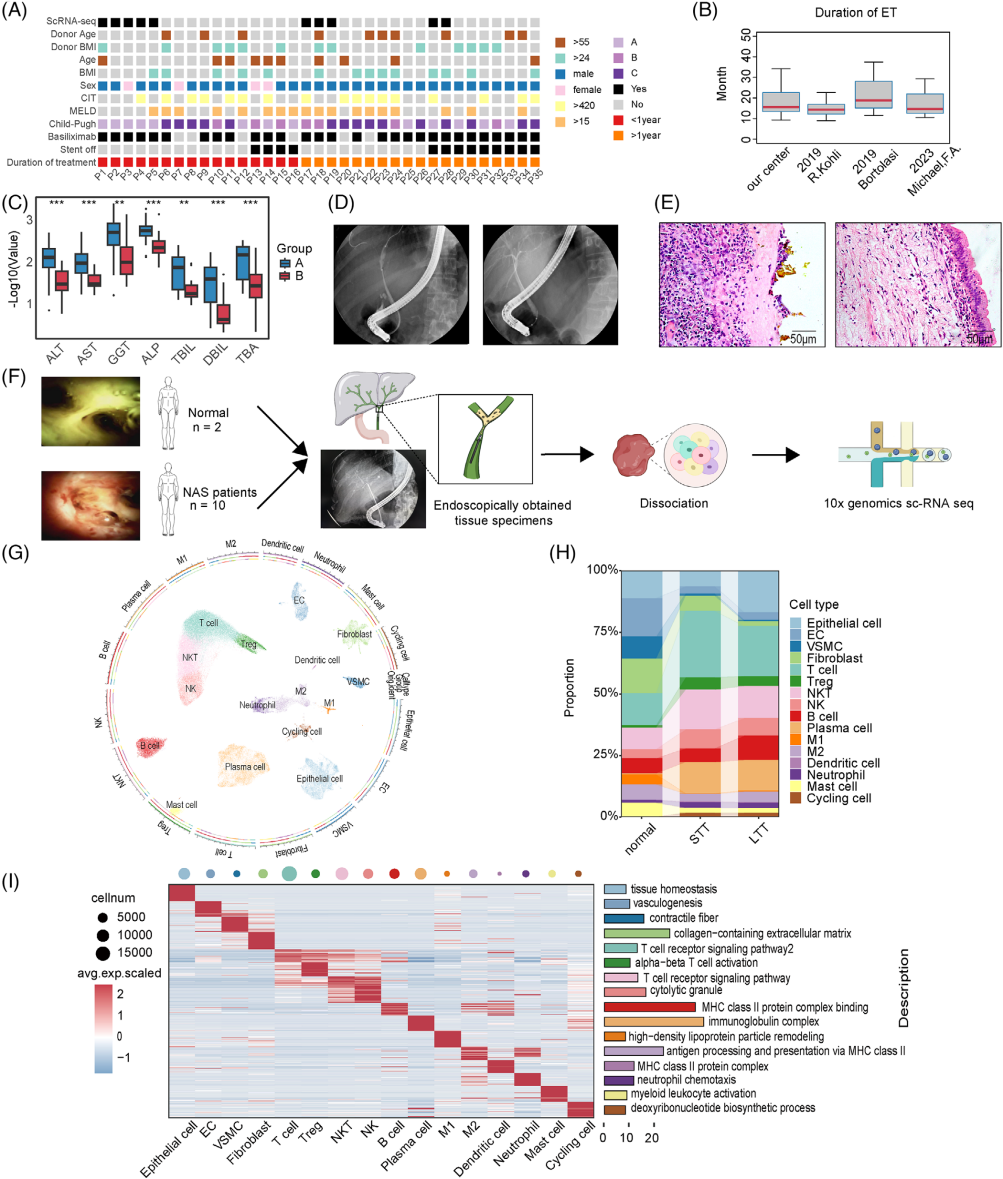
Cohort characteristics and scRNA‐seq of bile duct biopsies from NAS patients and donors. (A) Heatmap illustrating the clinical data of 35 NAS patients from our centre. (B) Box plots showing the average ET duration of NAS patients from four centres. (C) Box plots comparing ALT, AST, GGT, ALP, TBIL, DBIL, and TBA levels of NAS patients at the time of initial disease onset (group A) and after one year of ET (group B). NS, no significance; *P < 0.05; **P < 0.01; ***P < 0.001. (D) Cholangiographic presentations of posttransplant cholangiopathy in NAS patients at the time of initial onset (panel A) and one year after ET (panel B). (E) Histological images demonstrate different levels of biliary injury between the STT (panel A) and LTT (panel B) groups. (Panel A) A large amount of loss and damage of single‐layer columnar epithelial cells, as well as the appearance of acellular connective tissue beneath the epithelium, accompanied by a large number of infiltrating inflammatory cells. (Panel B) Mild damage, intact single‐layer columnar epithelium, and a small amount of infiltrating inflammatory cells. Scale bars = 50 µm. (F) Workflow of bile duct biopsy collection, dissociation and sequencing. (G) UMAP plots of 77 038 high‐quality single cells from normal (*n* = 2), STT (*n* = 5), and LTT groups (*n* = 5). (H) Bar plots showing the proportion of cell types in each group. (I) Heatmap showing the top 25 cell‐type‐specific genes (left). Representative Gene Ontology (GO) terms for marker genes (right). BMI, body mass index; CIT, cold ischemia time; ET, endoscopic treatment; MELD, model for end‐stage liver disease.

We summarized the demographics and clinical characteristics of 35 NAS patients treated at our centre (Figure 1A). Data from NAS patients with de‐stented in our centre and in other reported studies revealed that the mean ET duration of NAS was 13.3–19 months (Figure 1B).^14^, ^15^, ^16^ Additionally, NAS patients who have undergone ET for more than 1 year, demonstrated statistically significant improvement in liver function, along with radiological remission of stricture, following 1‐year of ET compared with the initial diagnosis (Figure 1C, D). We divided NAS patients into STT and LTT groups, using 1‐year of ET duration as the criterion. The cold ischemia time and the use of basiliximab showed no significant differences between the two groups (Table S4). In the STT group, there was observed destruction of biliary epithelial cells, extensive infiltration of inflammatory cells, bile stasis, and absence of nuclear connective tissue (Figure 1E). In contrast, the LTT group exhibited well‐preserved bile duct epithelial cell morphology and ductal structure, with minimal infiltration of inflammatory cells.

### 3.2 Transcriptomic landscape of the bile duct in NAS patients as defined by scRNA‐seq

To investigate the pathogenesis of NAS and the repair mechanism after long‐term ET, we conducted scRNA‐seq analysis of bile duct specimens from 10 NAS patients receiving ET (five STT and five LTT) and two normal bile duct specimens (Figure 1F).^17^ We have confirmed the histological and gene expression similarities between the extrahepatic hilar bile duct and common bile duct using rat experiments (Figure S1A–E). The biological samples from NAS patients receiving first‐time ET had a low viable cell rate and high spheroid formation rate, resulting in a low overall cell mass, making it difficult to meet the requirements for sequencing (Table S6). We sequenced a total of 77 038 cells and identified 25 distinct clusters using UMAP and graph‐based clustering (Figure 1G). These clusters were accurately annotated into 16 major cell types using established marker genes (Figure S1D, E). Based on specific marker genes, we identified the majority of cell types present in the biliary tissue, including epithelial cells (*KRT7*), endothelial cells (EC; *ERAMP2*), vascular smooth muscle cells (VSMC; *ACTA2*), fibroblasts (*COL3A1*), T cells (*CD3G*), T‐regulatory cells (Treg; *FOXP3*), natural killer T cells (NKT; *NKG7*), natural killer (NK) cells (*CD3G, NKG7*), B cells (*CD79A*), plasma cells (*JCHAIN*), M1 macrophages (M1; *CD68*), M2 macrophages (M2; *CD163*), dendritic cells (*IRF7*), neutrophils (*S100A8*), mast cells (*TPSB2*), and cycling cells (*MKI67*). Consistent with the H&E staining results, epithelial cells were more abundant in the LTT group than in the STT group (Figure 1H). We conducted gene set enrichment analysis (GSEA) using the top 25 cell‐type‐specific marker genes and illustrated the distinct functions of each cell type (Figure 1I). We defined the differences between the STT and normal groups as indicators of the cellular and genetic mechanism of NAS. The difference between the LLT and STT groups was regarded as evidence of the evolution induced by ET.

### 3.3 Gene expression across various cell types in NAS

**FIGURE 2:**
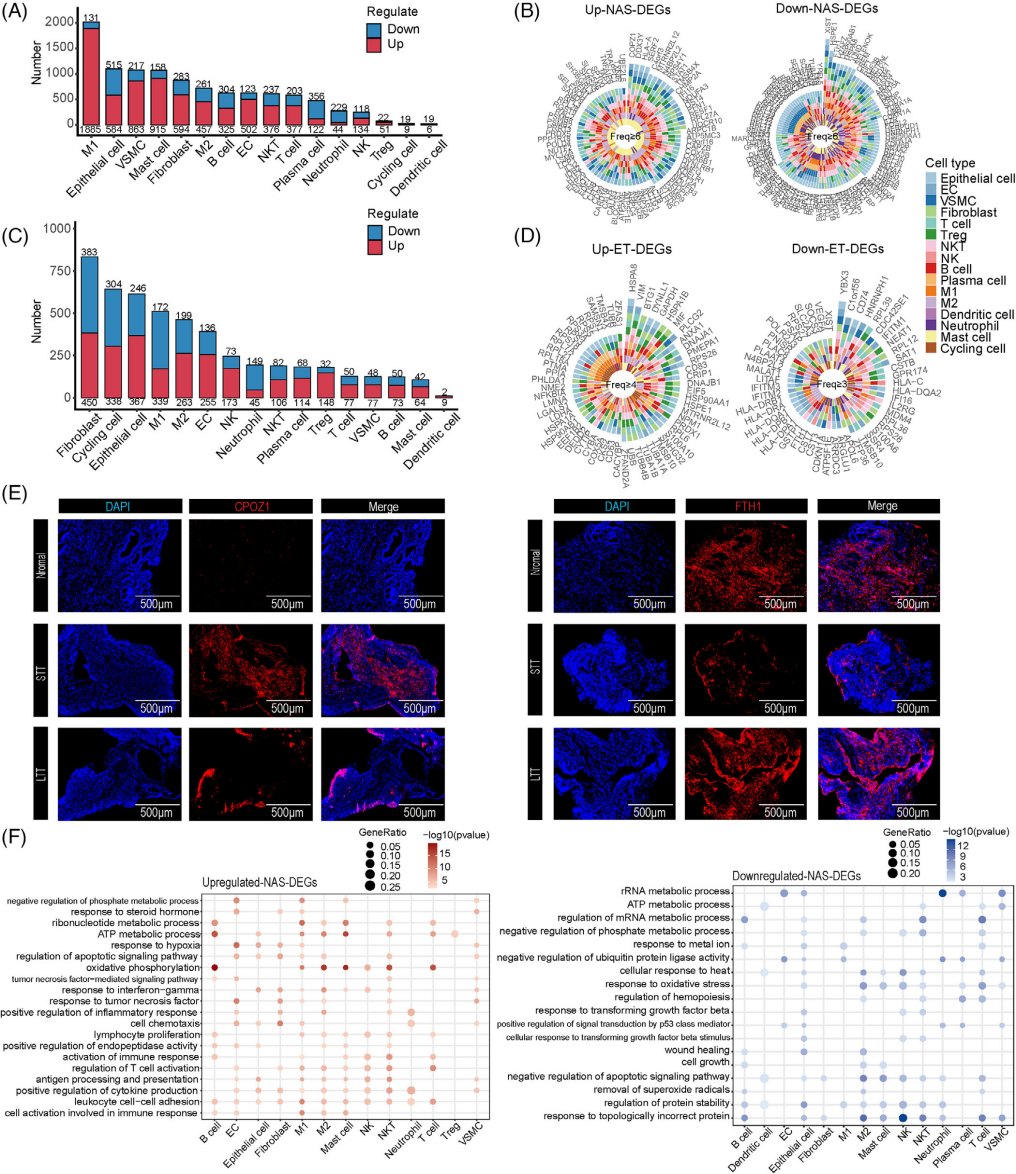
Effect of NAS and ET on all cell types. (A) Bar plots showing the number of NAS‐DEGs (upregulated and downregulated) in each cell type. (B) Plots showing the upregulated NAS‐DEGs (left) and downregulated NAS‐DEGs (right) shared by at least six cell types. (C) Bar plots showing the number of ET‐DEGs (upregulated and downregulated) in each cell type. (D) Plots showing the upregulated (left) and downregulated (right) ET‐DEGs shared by at least four and three cell types. (E) IF staining showing the expression of *COPZ1* (left) and *FTH1* (right) in bile duct tissue of the normal, STT, and LTT groups. Scale bars = 500 µm. (F) Dot plots summarizing the common biological pathways enriched among upregulated NAS‐DEGs (left) and downregulated NAS‐DEGs (right) in the major cell types. DEGs, differentially expressed genes.

To identify the changes in gene expression across various cell types of the bile duct in NAS, we identified differentially expressed genes (DEGs) from 16 distinct cell types within the normal and STT groups (Table S7). We defined “NAS‐DEGs” as upregulated or downregulated genes in the STT group compared to the normal group. We found more upregulated NAS‐DEGs than downregulated ones. M1 and epithelial cells had a higher number of NAS‐DEGs than other cell types. (Figure 2A). To reveal the commonality of NAS‐DEGs across various cell types, we identified 80 consistently upregulated NAS‐DEGs and 112 consistently downregulated NAS‐DEGs that were present in at least six cell types (Figure 2B). COPZ1, a top upregulated NAS‐DEG, was positively correlated with cellular hypoxia (4) and showed increased expression in the STT group compared to the normal group, as confirmed by immunofluorescence (IF). Conversely, FTH1, an important antiferroptotic factor,^18^ was downregulated in the STT group compared with the normal group, which was also confirmed by IF (Figure 2E) and reverse transcription polymerase chain reaction (RT‐qPCR; Figure S2D).

Through GSEA of upregulated NAS‐DEGs, we discovered that immune‐ and inflammation‐related pathways were consistently activated across multiple cell types. The response to hypoxia and apoptosis pathways was mainly upregulated in nonimmune cells, while ATP metabolism and oxidative phosphorylation were mainly upregulated in immune cells (Figure 2F). After performing GSEA on downregulated NAS‐DEGs, we found common suppression of negative regulation of the apoptotic signalling pathway and regulation of protein stability pathways across multiple cell types; we also found downregulation of the response to transforming growth factor‐beta and wound healing pathways in epithelial cells. These findings suggested that the nonimmune cells in the bile duct tissue of NAS patients were in a hypoxic state and that the overall cells were in an inflammatory and immune microenvironment.

### 3.4 Reversal of NAS‐induced gene expression changes by ET

We explored the evolution induced by ET by comparing the DEGs between the LTT group and the STT group and defined the DEGs as “ET‐DEGs” (Table S8). Overall, the number of ET‐DEGs was less than that of NAS‐DEGs (Figure 2C). To reveal the commonality of ET‐DEGs across various cell types, we identified upregulated genes in at least four cell types and downregulated genes in at least three cell types (Figure 2D). The expression of VIM, a top‐upregulated ET‐DEG, was lower in the STT group than in the LTT group (Figure S2A).

**FIGURE 3:**
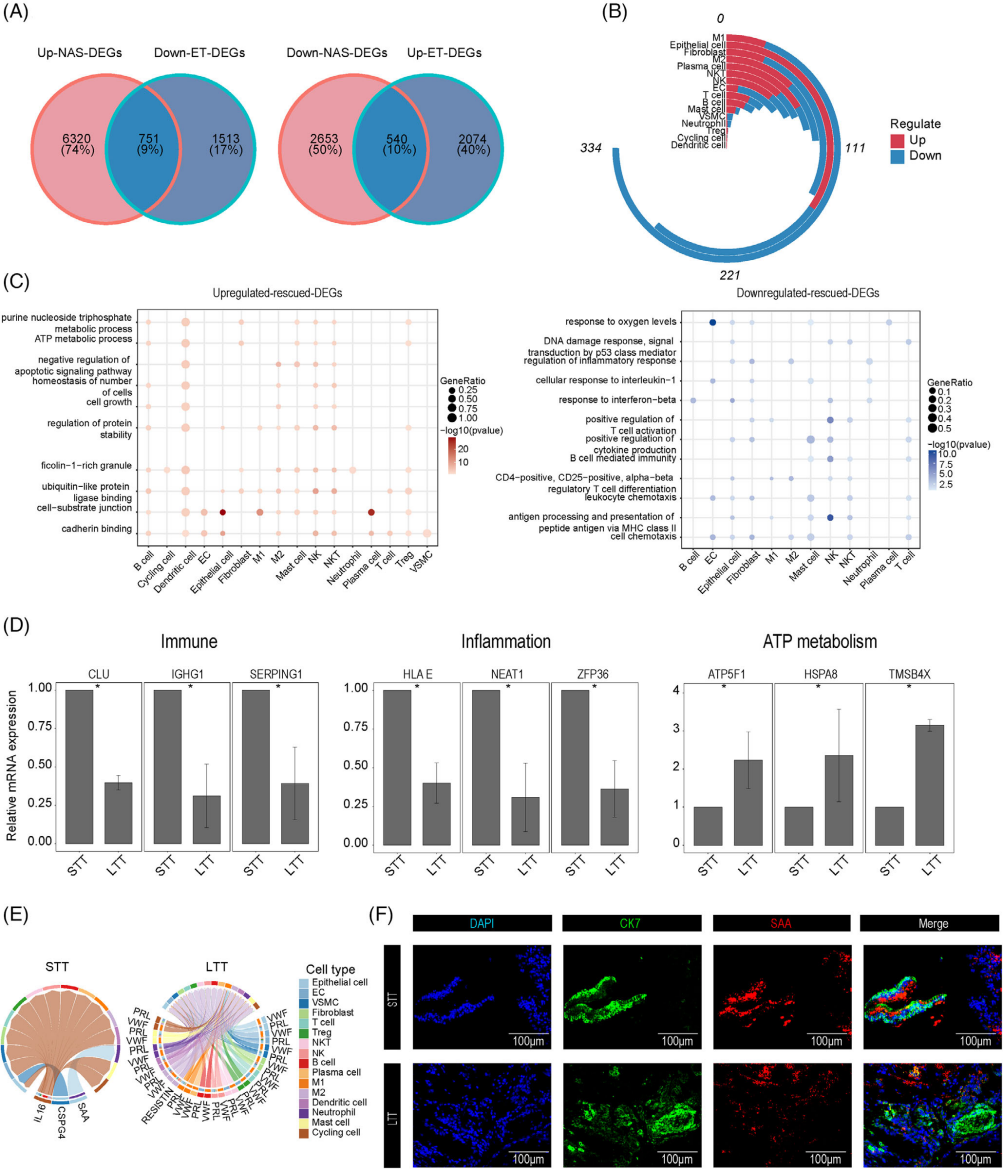
Evolution in all cell types due to rescued genes. (A) Venn diagram showing the number of total downregulated rescued genes (left) and upregulated rescued genes (right). (B) Plots showing the number of rescued genes (upregulated and downregulated) in all cell types. (C) Dot plots summarizing common biological pathways enriched among upregulated (left) and downregulated (right) rescued genes in major cell types. (D) Bar plots showing the relative mRNA expression levels of immune, inflammatory and ATP metabolic pathway‐related genes detected by RT‐qPCR between the STT and LTT groups. NS, no significance; *P < 0.05; **P < 0.01; ***P < 0.001. (E) Chord plots showing cell‒cell communication mediated by the SAA, CSPG4, and IL−16 pathways across all cell types in the STT group (left), and the VWF, PRL, and RESISTIN pathways across all cell types in the LTT group (right). (F) IF staining for SAA, CK7, and DAPI in bile duct tissue of the STT and LTT groups. Scale bars = 100 µm.

Next, we investigated the effects of ET on NAS‐DEGs in all cell types. During ET, the downregulated NAS‐DEGs showed an increase in expression level, while the upregulated NAS‐DEGs showed a decrease in expression level; these genes were collectively referred to as “rescued genes”. By comparison, we obtained 540 upregulated rescued genes and 789 downregulated rescued genes from all cell types (Figure 3A). We discovered that M1 and epithelial cells had more restored genes than other cell types (Figure 3B). Separate GSEA of up‐ and downregulated rescued genes revealed that downregulated rescued genes were enriched mainly in immune‐ and inflammation‐related pathways (Figure 3C). The upregulated rescued genes were enriched in metabolic pathways and biological processes such as ATP metabolism and cell‐substrate junctions during NAS. RT‐qPCR experiments confirmed significantly higher expression of inflammation pathway‐related genes (*HLAE*, *NEAT1*, *ZFP36*) and immune pathway‐related genes (*GLU*, *IGHG1*, *SERPING1*) in the STT group than in the LTT group. ATP metabolic pathway‐related genes (*ATP5F1D*, *HSPA8*, *TMSB4X*) exhibited opposite expression patterns (Figure 3D).

We utilized the CellChat package to assess the potential communication among all three groups of cell types.^19^ We identified 125 potential signalling pathways in the LTT group (Figure S3A, B; Table S9). By comparing the overall signalling pathways across all three groups, we found that the SAA, IL−16, and CSPG4 pathways were uniquely present in the STT group and that the PRL, VWF, and RESISTIN pathways were uniquely present in the LTT group (Figure 3E). The STT group showed a significant increase in the number of *SAA* ^+^ ck7^+^ cells, while the LTT group exhibited a decrease (Figure 3F). Epithelial cells output the most signals in the SAA, IL−16, and CSPG4 pathways in the STT group, while neutrophils received the most signals (Figure S3C, D). These findings indicate that in NAS, the increase in cell‐to‐cell SAA and IL−16 signals exacerbates the inflammatory response, especially in epithelial cells and neutrophils.

### 3.5 scRNA‐seq analysis revealed a reduction in the proportion of epithelial cells in NAS

**FIGURE 4:**
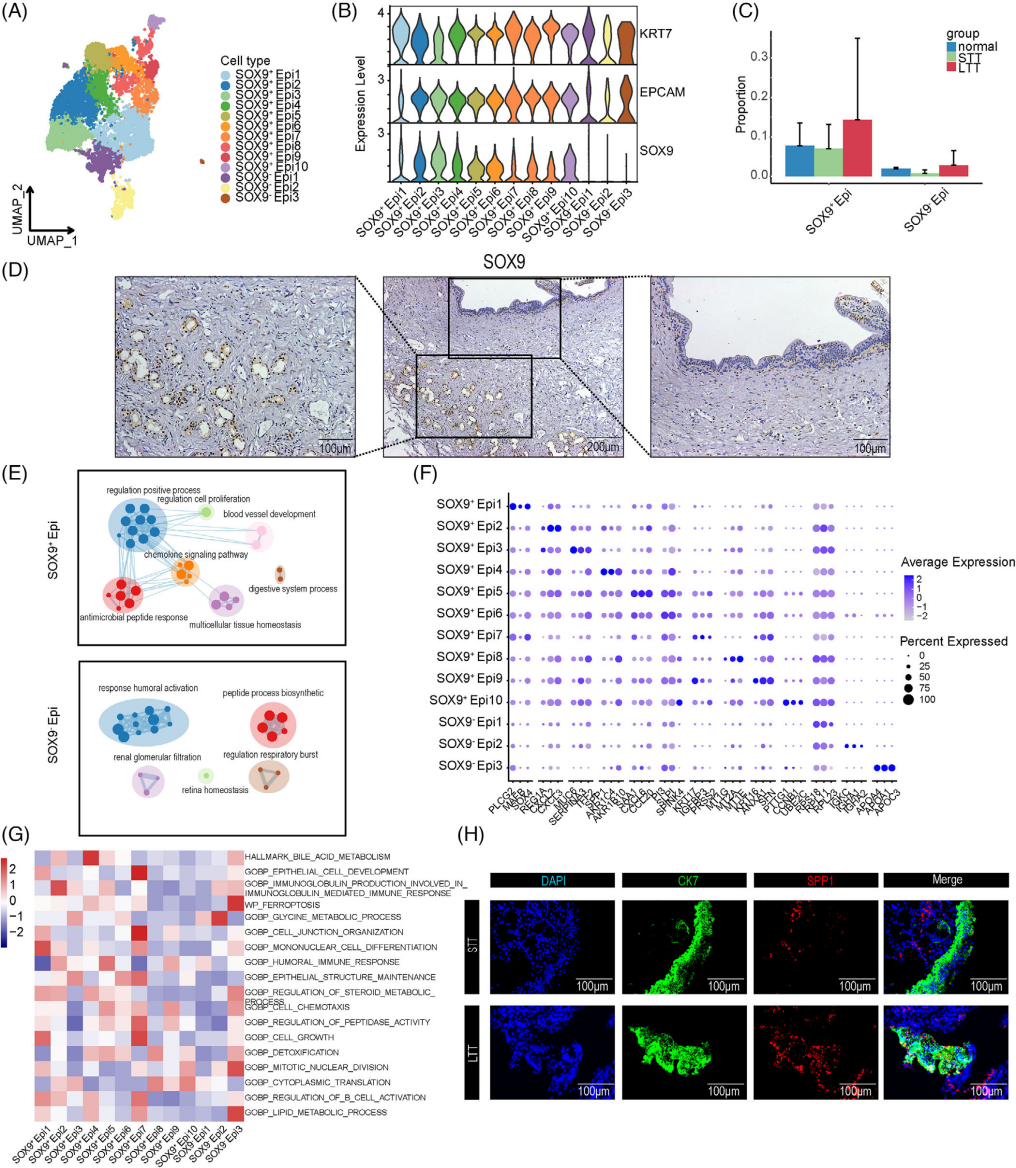
Subclusters of epithelial cells. (A) UMAP plots of 13 colour‐coded epithelial cell subclusters. (B) Violin plots showing the expression of *KRT7*, *EPCAM*, and *SOX9* in distinct epithelial cell subclusters. (C) Bar plots showing the overall proportion of three groups of *SOX9^+^* epithelial cells and *SOX9* ^−^ epithelial cells. (D) Immunohistochemical staining showing the expression of *SOX9* in the bile duct tissue of donors. Scale bars = 100 and 200 µm. (E) Network graph illustrating representative GO terms and pathways of *SOX9^+^* epithelial cells (above) and *SOX9^−^* epithelial cells (below). This plot was created with Cytoscape. (F) Dot plots showing marker genes for 13 distinct cell types. (G) Heatmap showing the functional assignment of each epithelial cell subcluster. (H) IF staining for *SPP1*, *CK7*, and *DAPI* in bile duct tissue. Scale bars = 100 µm.

Focused subclustering analysis of epithelial cells identified 13 subclusters (Figure 4A). Based on the expression of *SOX9*, the cells were divided into *SOX9* ^+^ and *SOX9* ^–^ epithelial cells (Figure 4B). Compared with *SOX9* ^−^ epithelial cells, *SOX9* ^+^ epithelial cells expressed higher levels of genes related to stemness and proliferation, such as *SOX4*, *KLF4*, *KLF5*, *PROM1*, *CD44*, and *PCNA* (Figure S4A). We found that the proportions of *SOX9^+^* and *SOX9^–^* epithelial cells were higher in the normal and LTT groups than in the STT group (Figure 4C). Immunohistochemistry (IHC) confirmed that *SOX9* expression was concentrated mainly in the peribiliary glands (Figure 4D). *SOX9^+^* cells also had high expression of *PCNA* (Figure S4C). According to GSEA, *SOX9^+^*, and *SOX9^–^* epithelial cells were both involved in peptide processing (Figure 4E). Additionally, *SOX9^+^* epithelial cells helped to regulate cell proliferation, blood vessel development, and multicellular tissue homeostasis. The LTT group had a higher number of *SOX9^+^* cells than the STT group (Figure S4D). The decrease in *SOX9^+^* epithelial cells during NAS resulted in impaired proliferation ability and homeostasis of epithelial and ECs.

By analyzing the proportions of subclusters, we found that *SOX9* ^−^ Epi3 cells, which were enriched in the STT group, had high expression of ferroptosis‐related genes (*MUC6*, *SERPINA3*, *TFF2*; Figure 4F). The proportions of *SOX9^+^* Epi4, *SOX9^+^* Epi8, and *SOX9^–^* Epi1 cells were effectively restored after ET (Figure S4B). *SOX9* ^+^ Epi4 cells were enriched with steroid metabolic genes (*SPP1*, *AKR1C4*, *AKR1B10*), *SOX9* ^+^ Epi8 cell were enriched with detoxification genes (*MT1G*, *MT2A*, *MT1E*), and *SOX9* ^–^ Epi1 cells were enriched with cytoplasmic translation genes (*RPS18*, *RPL11*, *RPL23*). Through gene set variation analysis (GSVA), we found that *SOX9^+^* Epi5 cells, which were enriched in the STT group, mainly mediated humoral immunity, and that *SOX9^+^* Epi4 cells played a major role in bile acid metabolism (Figure 4G). Through IF, we confirmed that the number of *SPP1^+^ CK7^+^* cells increased after ET (Figure 4H). These results suggested that NAS and ET were correlated with alterations in the proportion and phenotype of epithelial cell subclusters.

Through additional analysis, we found that upregulated ET‐DEGs in epithelial cells functioned mainly in metabolic process‐related biosynthesis and small subunit biogenesis, while downregulated ET‐DEGs in epithelial cells participated primarily in exogenous peptide antigen and response endogenous stimulus (Figure S4E). CellChat was used to calculate the potential communication among all epithelial cell subclusters. In the LTT group, a total of 85 possible intercellular communications were identified (Figure S5A). By comparing the overall cell communication among the three groups of epithelial‐cell subclusters, we discovered that a total of 16 communications were reinstated with ET. These communications included growth factor‐related pathways, such as those involving VEGF, IGF, and HGF (Figure S5B). Therefore, we examined all growth factor‐related communications and determined that the three restored communication pathways were present in multiple cell types (Figure S5C). Through IF, we confirmed that the number of *MET1^+^ CK7^+^* cells increased after ET (Figure S5D).

### 3.6 NAS is characterized by a reduction in the proportion of EC and an increase in fibroblasts

**FIGURE 5:**
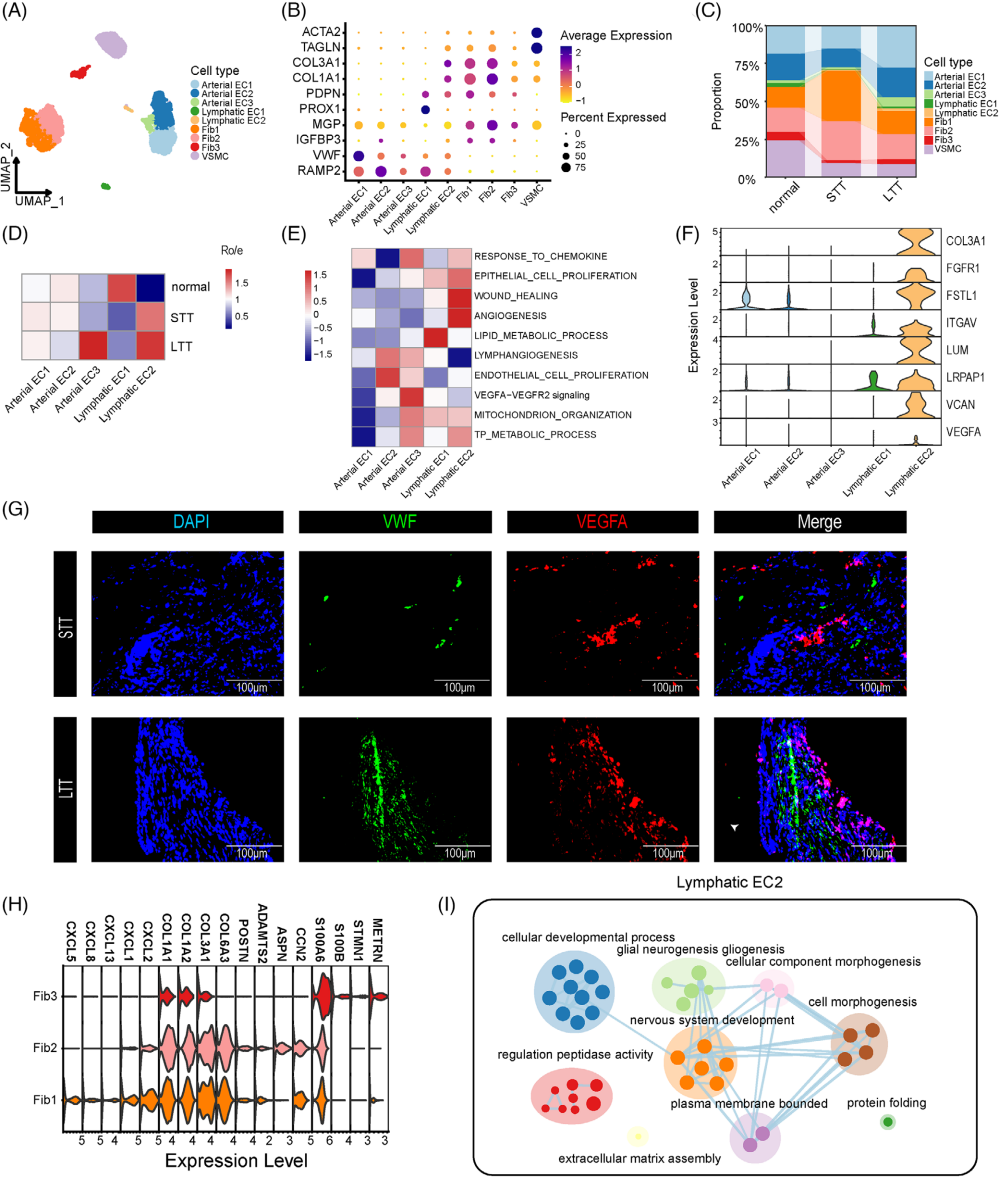
Subclusters of mesenchymal cells. (A) UMAP plots of nine colour‐coded mesenchymal cell subclusters. (B) Dot plots showing marker genes for nine distinct cell types. (C) Bar plots showing the overall fraction of the three groups among all mesenchymal cell subclusters. (D) Heatmap showing preferential enrichment of five endothelial cell subclusters across three groups. (E) Heatmap showing the functional assignment of each mesenchymal cell subcluster. (F) Violin plots showing the expression of *VEGFA*, *VEGFB*, *VEGFC*, *VEGFD*, *FLT1*, *FLT4*, and *KDR* in distinct endothelial cell subclusters. (G) IF staining for *VEGFA* and *VWF* in bile duct tissue of the STT and LTT groups. Scale bars = 100 µm. (H) Violin plots showing the expression of specific marker genes in distinct fibroblast subclusters. (I) Network graph illustrating representative GO terms and pathways of lymphatic EC2. This plot was created with Cytoscape.

We clustered EC, fibroblasts, and VSMC, using classic marker genes to divide those cells into nine subclusters, including three subclusters of arterial EC, two subclusters of lymphatic EC, three subclusters of fibroblasts, and one subcluster of VSMC (Figure 5A, B). We found that the proportions of EC and fibroblasts were restored during ET (Figure 5C). The proportion of lymphatic EC1 cells was decreased in the STT group and partially recovered in the LTT group and the proportion of lymphatic EC2 cells continued to increase during ET. Arterial EC3 cells were significantly increased in the LTT group (Figure 5D). Through GSVA, we found that lymphatic EC1 played a significant role in lipid metabolism, while lymphatic EC2 played a major role in wound healing and angiogenesis (Figure 5E). Additionally, arterial EC3 was significantly involved in the VEGFA‐VEGFR2 signalling pathway. The expression of genes related to angiogenesis pathways was significantly higher in lymphatic EC2 cells than in other cell types (Figure 5F). Through IF staining, we verified that the number of *VWF^+^VEGFA^+^* cells in the LTT group was higher than that in the STT group (Figure 5G). This difference represented a decrease in the size of the lipid metabolism EC subcluster in the NAS and a sustained increase in the size of the angiogenesis EC subcluster following ET.

By examining specific marker genes of fibroblast subclusters, we defined Fib1 as a subcluster of proinflammatory fibroblasts expressing chemokine genes (*CXCL5*, *CXCL8*, *CXCL13*, *CXCL1*, *CXCL2*), and Fib2 as a subcluster of mesenchymal fibroblasts expressing collagen genes (*COL1A1*, *COL1A2*, *COL3A1*, *COL6A3*) (Figure 5H).^20^ Additionally, we found that the Fib3 subcluster exhibited high expression of axonogenesis‐related genes (*S100A6*, *S100B*, *STMN1*, *METRN*), which was consistent with previous literature.^21^ The proportions of the Fib1 and Fib2 subclusters were increased in the STT group, but that of Fib3 was decreased. GSEA showed a significant increase in gene expression in pathways involved in axonogenesis, and glial neurogenesis gliogenesis, indicating a potential neuromodulation function of the Fib3 subcluster during NAS and ET processes (Figure 5I). These results suggested that NAS accompanied the progression of bile duct fibrosis.

### 3.7 A phenotypic shift in macrophages occurs during NAS and ET

**FIGURE 6:**
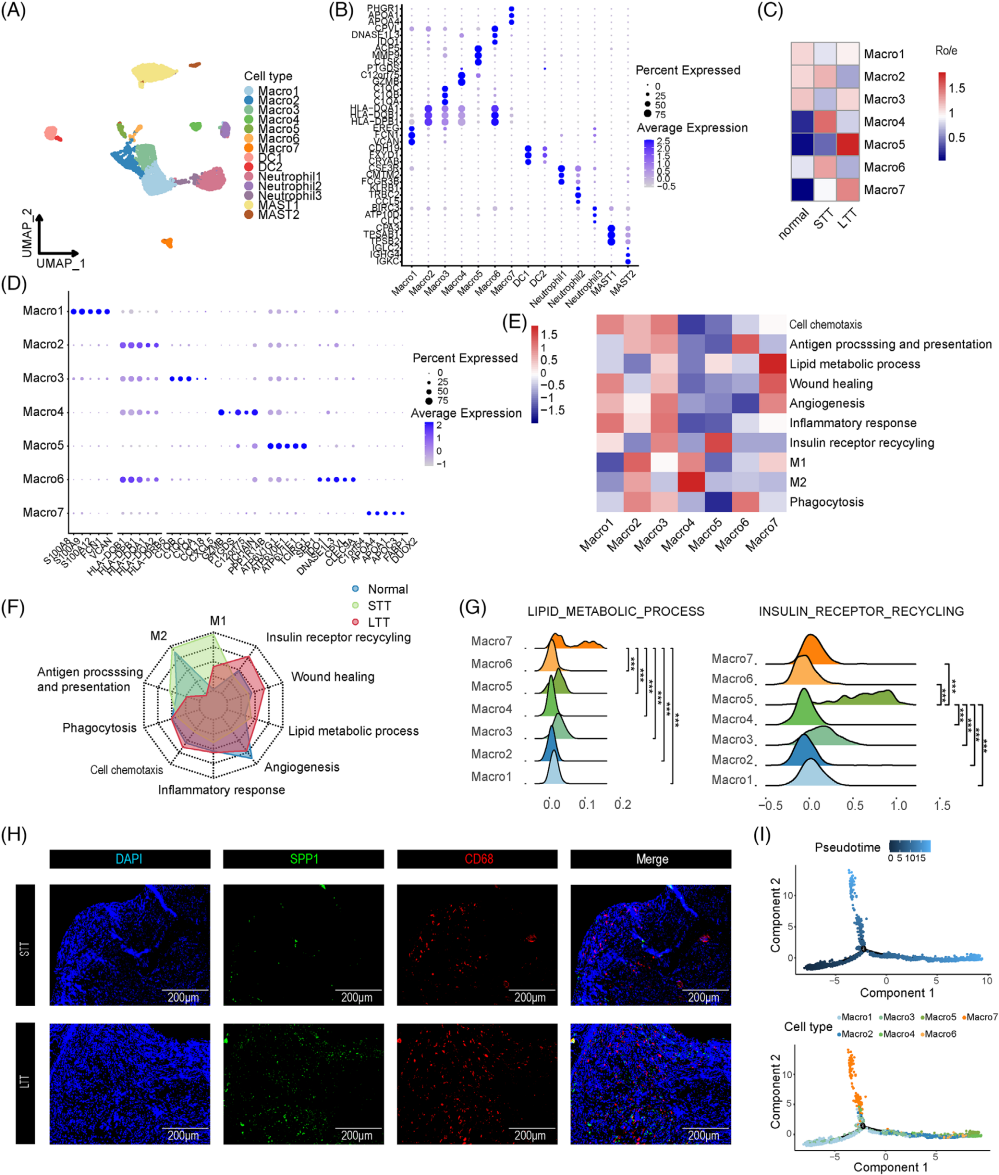
Subclusters of myeloid cells. (A) UMAP plots of 14 colour‐coded myeloid cell subclusters. (B) Dot plots showing marker genes for 14 distinct cell types. (C) Heatmap showing preferential enrichment of seven macrophage subclusters across three groups. (D) Dot plots showing marker genes for seven distinct macrophage subclusters. (E) Heatmap showing the functional assignment of each macrophage subcluster. (F) Radar plots showing the functional assignment of each group. (G) Ridge plots showing the lipid metabolic process (left) and insulin receptor recycling (right) scores of 7 macrophage subclusters. NS, no significance; *P < 0.05; **P < 0.01; ***P < 0.001. (H) IF staining for *SPP1*, *CD68*, and *DAPI* in bile duct tissue of the STT and LTT groups. Scale bars = 200 µm. (I) Pseudotrajectory of macrophage subclusters (above) and cell type transition (below).

Next, we merged M1, M2, dendritic cells, neutrophils, and mast cells, and identified fourteen major subsets (Figure 6A). Each cell type expressed different marker genes at high levels (Figure 6B). Macrophages are essential in LT because they clear necrotic tissue, regulate inflammation, promote graft repair and regeneration, and participate in immune regulation and the antiviral immune response.^22^ We obtained a total of seven clusters of macrophages, among which Macro1 showed high expression of S100A‐family genes (*S100A8*, *S100A9*, *S100A12*, *FCN1*, *VCAN*), indicating that it was a proinflammatory macrophage cluster. Macro2 was more prevalent in the STT group and exhibited antibody presentation characteristics (*HLA–DQB1*, *HLA–DPB1*, *HLA–DQA1*, *HLA–DQA2*, *HLA–DRB5*). Complement C1q appeared as a marker for tolerant and immunosuppressive macrophage populations in Macro3. Macro4 showed high expression of a T‐cell proliferation suppression gene (*GZMB*). Macro5 was more prevalent in the LTT group than in the other groups and played a role in insulin receptor recycling. Macro6 showed high expression of the genes *IDO1* and *DNASE1L3*, while Macro7 was enriched in the LTT group and played a role in lipid metabolism (*APOA4*, *APOA1*, *APOC3*, *FABP1*), while also showing high expression of dual oxidase 2 (*DUOX2*) (Figure 6C, D).

We selected relevant pathways and calculated the scores of each pathway in different clusters of macrophages and groups. We found that the STT group was enriched mainly in M1, M2, macrophage differentiation and antigen processing and presentation pathways, while the LTT group tended to be enriched in lipid metabolism, insulin receptor recycling and wound repair pathways (Figure 6E, G). Through IF staining, we verified that the number of *SPP1^+^CD68^+^* cells was higher in the LTT group than in the STT group (Figure 6H). By analyzing the trajectory of macrophage subclusters, we found that a differentiation process from Macro1 and Macro3 to Macro5 and Macro7 cells occurred (Figure 6I). This finding indicated that ET led to a transition in the cell phenotype from proinflammatory to metabolic.

### 3.8 NAS results in explosive infiltration of plasma cells

**FIGURE 7:**
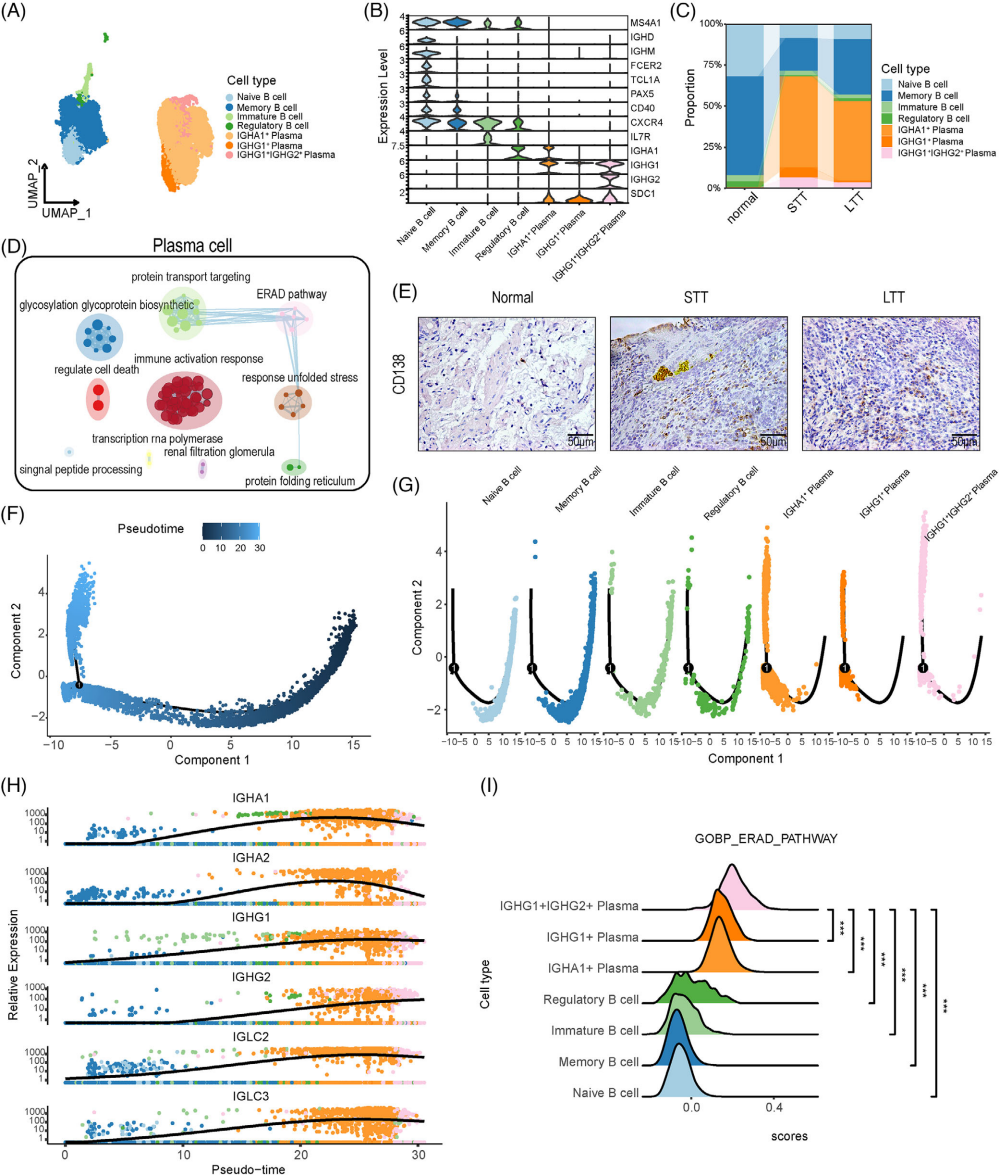
Subclusters of B‐ and plasma cells. (A) UMAP plots of seven colour‐coded myeloid cell subclusters. (B) Violin plots showing marker genes for seven distinct cell types. (C) Bar plots showing the proportion of cell types in each group. (D) Network graph illustrating representative GO terms and pathways of plasma cells. This plot was created with Cytoscape. (E) Immunohistochemical staining showing the expression of *CD138* in bile duct tissue of the normal, STT, and LTT groups. Scale bars = 50 µm. (F) Pseudotrajectory of all B‐ and plasma‐cell subclusters. (G) Pseudotrajectory of cell type transition. (H) Expression patterns of some immunoglobulin‐related genes under all B‐ and plasma‐cell subclusters. (I) Ridge plots showing the ERAD pathway scores of all B‐ and plasma‐cell subclusters. NS, no significance; *P < 0.05; **P < 0.01; ***P < 0.001.

We merged B‐ and plasma cells and performed a reductive clustering to identify seven distinct subclusters (Figure 7A). Based on specific marker genes, these subclusters were defined as naïve B cells, memory B cells, immature B cells, regulatory B cells, and three subclusters of plasma cells (Figure 7B). We found that the normal group consisted primarily of naïve B cells and memory B cells, with very few plasma cells. However, the STT group showed a significant increase in the proportion of plasma cells, while the LTT group had a slight decrease (Figure 7C). GSEA of all plasma cells revealed that they participated mainly in the immune activation response, unfolded protein response (UPR), and endoplasmic reticulum‐associated degradation (ERAD) pathway (Figure 7D). IHC analysis revealed a substantial elevation in the number of plasma cells in the bile duct tissue of the NAS group compared with that of the normal group (Figure 7E).

Using the Monocle2 package to construct differentiation trajectories for all B‐ and plasma cells, we found that the initial point was composed predominantly of B‐cell subclusters, which differentiated toward plasma cells, with the most terminal cluster being the *IGHG1^+^ IGHG2^+^* plasma cells (Figure 7F, G). Analysis of the expression patterns of related immunoglobulin genes showed significant upregulation from the B‐cell subclusters to the plasma cell subclusters, with expression peaking in the *IGHA1^+^* plasma cells and decreasing in the *IGHG1^+^ IGHG2^+^* plasma cells (Figure 7H). The ERAD pathway scores of the *IGHG1^+^ IGHG2^+^* plasma cell subclusters were the highest (Figure 7I). Scoring of immunoglobulin‐related gene expression in all three groups of cells revealed that the STT group had significantly higher scores than the other two groups (Figure S6A). This suggests that explosive infiltration of plasma cells may be one of the mechanisms underlying NAS.

### 3.9 Dysfunction of T‐ and NK cells in NAS

**FIGURE 8:**
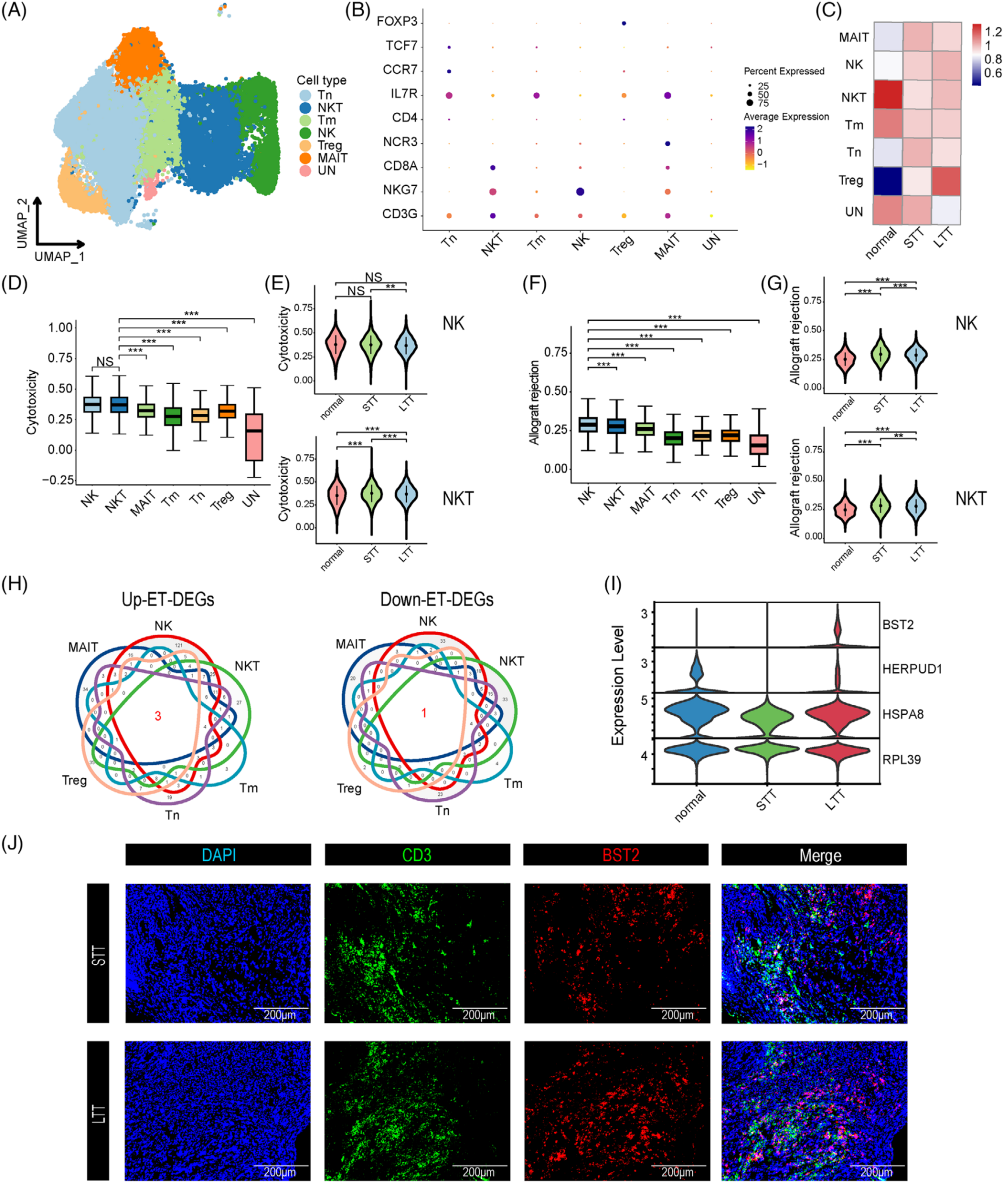
Subclusters of T‐ and NK cells. (A) UMAP plots of seven colour‐coded T‐ and NK‐cell subclusters. UN, unknown. (B) Dot plots showing marker genes for seven distinct cell types. (C) Heatmap showing the preferential enrichment of seven T‐ and NK subclusters across the three groups. (D) Box plots showing cytotoxicity scores for T‐ and NK‐cell subclusters, obtained by comparing NKT cells with other T‐ and NK‐cell subclusters. NS, no significance; *P < 0.05; **P < 0.01; ***P < 0.001. (E) Violin plots comparing cytotoxicity scores for NK (above) and NKT (below) cells among the normal, STT, and LTT groups. NS, no significance; *P < 0.05; **P < 0.01; ***P < 0.001. (F) Box plots showing rejection scores for T‐ and NK‐cell subclusters, obtained by comparing NKT cells with other T‐ and NK‐cell subclusters. NS, no significance; *P < 0.05; **P < 0.01; ***P < 0.001. (G) Violin plots comparing the rejection scores for NK (above) and NKT (below) cells among the normal, STT, and LTT groups. NS, no significance; *P < 0.05; **P < 0.01; ***P < 0.001. (H) Venn diagram showing the upregulated (*BST2*, *HERPUD1*, and *HSPA8*) (left) and downregulated (*RPL39*) (right) ET‐DEGs that are commonly expressed in six T‐ and NK‐cell subclusters. (I) Violin plots showing the expression of *BST2*, *HERPUD1*, *HSPA8*, and *RPL39* in six T‐ and NK‐cell subclusters of three groups. (J) IF staining for *BST2* and *CD3* in bile duct tissue of the STT and LTT groups. Scale bars = 200 µm.

Next, we merged T cells, Treg, NKT cells, and NK cells, and identified seven major subsets, including naïve T (Tn), memory T (Tm) and Treg, mucosal‐associated invariant T, and NK‐ and NKT‐cell subsets (Figure 8A). Each cell type was labelled using typical marker genes, but cluster 7 lacked markers and was named “unknown (UN)” (Figure 8B). We found an enrichment of Treg in the LTT group (Figure 8C). We calculated the cytotoxicity, allograft rejection, and negative regulation of adaptive immune response pathway scores for all cell types and groups. NK and NKT cells had significantly higher cytotoxicity pathway scores than other cell types. Additionally, the STT group had significantly higher scores for NK and NKT cells in this pathway than the normal and LTT groups (Figure 8D, E). Similarly, NK and NKT cells were revealed to play an important role in allograft rejection (Figure 8F, G). In terms of negative regulation of the adaptive immune response, all major T cells were equally important (Figure S6B). Through intergroup comparisons, we found that all types of T‐cell‐mediated immune regulation were stronger in the normal and LTT groups than in the STT group (Figure S6C). By searching for the ET‐DEGs in the main T‐cell types, we found that the genes *BST2*, *HERPUD1*, and *HSPA8* were upregulated in all T cells after ET, while the *RPL39* gene was downregulated (Figure 8H, I). Through IF staining, we verified that the number of *BST2^+^ CD3^+^* cells was higher in the LTT group than in the STT group (Figure 8J). These results indicated that the distribution of NK and NKT cells in the STT group was dysfunctional and abnormal, providing a resource for further investigation of NAS immunotherapy.

## 4 DISCUSSION

NAS, one of the most common postoperative complications after LT, severely affects the survival and prognosis of patients.^2^, ^3^ However, the lack of animal models and difficulty in obtaining clinical specimens have severely hindered research on the mechanisms of NAS. Our study used scRNA‐seq to analyze the cellular composition and transcriptomic landscape of NAS and its evolution during ET to provide insights for improved NAS prevention and treatment. To our knowledge, our study is the first to report the cellular composition and transcriptomic landscape of NAS and its evolution during ET, further providing insights into the prevention and treatment of NAS.

The bile duct tissues of NAS patients exhibit a gradual onset of epithelial injury due to chronic ischemia and hypoxia that manifests only after a significant delay, typically within the first 3 to 6 months following transplantation, but potentially even later.^7^ In our comparison between the STT and normal groups, we observed not only that epithelial cells are in a state of prolonged response to hypoxia, but also that mesenchymal cells, such as EC, fibroblasts, and VSMC, exhibit a stronger response to hypoxic conditions than most immune cells. However, the oxidative phosphorylation and ATP metabolism process pathways of most immune cells are significantly upregulated, which ensures their normal function with a sufficient energy supply.^23^ This demonstrates the heterogeneity of the hypoxia response and energy metabolism between nonimmune and immune cells. The significant reduction in the proportion of EC exacerbates the ischemic and hypoxic conditions of the tissue. Almost all cell types exhibited upregulation of the *COPZ1* gene, which is associated with hypoxia.^24^ We also discovered that the wound healing ability, response to transforming growth factor‐beta and negative regulation of phosphate metabolism functions of the epithelial cells in the STT group were inhibited. Importantly, we found that after ET, the response of these cells to oxygen levels was downregulated, which played a crucial role in the recovery of the functions of parenchymal and mesenchymal cells.^25^ The restoration of cell‐substrate junctions in various cell types is also a significant finding, as it suggests improved efficacy of intracellular communication and cooperation to restore tissue function and promote healing.^26^

We observed the upregulation of inflammation‐related pathways, including the tumour necrosis factor and interferon‐gamma pathways, in multiple cell types in the STT group compared with the normal group. Additionally, NAS is due in part to chronic inflammation.^27^ By assessing cell‒cell communication, we found that the SAA and IL‒16 pathways appeared independently in the STT group. As these molecules are proinflammatory adipokines and cytokines, respectively,^28^, ^29^ their presence indicates an increase in inflammation in the STT group. However, following ET, significant downregulation of inflammation‐related pathways, including the interleukin‐1 and interferon‐beta pathways, was observed. This emphasizes the crucial role of inflammation in the progression of NAS and highlights the potential therapeutic benefits of ET in reducing inflammation in NAS patients.

Simultaneously, we observed that the NAS complex upregulates immune‐related pathways, including antigen processing and presentation, lymphocyte proliferation, and regulation of T‐cell activation, in multiple cell types. The cytotoxicity and allograft rejection scores of NK and NKT cells in the STT group were significantly higher than those in the other groups. Following ET, significant reductions in the activation of these pathways were observed, indicating a crucial complement to the role of the immune system in NAS. And we found that the genes *BST2* and *HERPUD*1 were upregulated in all T cells after ET. *BST2* upregulation can activate EGFR to promote epithelial cell growth, while also enhancing the body's resistance to viruses.^30^ HERPUD1 plays a vital role in cell proliferation by inhibiting apoptosis, influencing the cell cycle, and inhibiting the EMT, PI3K/AKT/mTOR, and p38MAPK pathways.^31^ Under the interference of multiple mixed factors, the apoptotic pathway was upregulated in multiple cell types in the STT group and downregulated in multiple cell types in the LTT group. In addition, we found that the expression of the *FTH1* gene was inhibited in multiple cell types of the STT group. *FTH1* plays a crucial antiferroptotic role, and its downregulation may lead to the progression of ferroptosis in multiple cell types.^18^ Consistent with previous studies, we also observed the absence of biliary epithelial cells and peribiliary glands in pathological sections of NAS patients.^32^ Therefore, inhibiting ferroptosis in peribiliary glands and epithelial cells may be a potential therapeutic option.

To investigate the changes in subclusters of biliary epithelial cells in NAS, we continued to perform subcluster analysis of the epithelial cells. The *SOX9^+^* epithelial cell group expressed some genes related to stemness and proliferation, such as *SOX4*, *KLF4*, *KLF5*, *PROM1*, *CD44*, and *PCNA*. Consistent with previous findings, we speculate that *SOX9^+^* epithelial cells are more similar to peribiliary glands than mature epithelial cells.^33^ IHC revealed that p eribiliary glands expressed higher levels of *SOX9* than mature epithelial cells. Furthermore, there was a significant reduction in the number of *SOX9^+^* epithelial cells in the STT group compared with the normal group, and some recovery was observed following ET. As the peribiliary glands represent a stem cell niche, their health and abundance play a crucial role in the proliferation and maintenance of biliary epithelial cells.^34^, ^35^ We found that *SOX9^+^* epithelial cells are also important participants in vascular development and multicellular homeostasis. The enriched *SOX9^+^* Epi5 cells in the STT group mainly mediated humoral immunity, while *SOX9^−^* Epi3 cells had high expression of ferroptosis‐related genes. Importantly, we observed that *SOX9^+^* Epi4 cells participated in steroid and bile acid metabolism, and were restored following ET. Although biliary epithelial cells represent only 3−5% of the total liver cells, they contribute up to 30% of daily bile production in humans.^36^ Steroid hormones, as important components of bile, are critical for the maintenance of normal digestive, absorptive, and metabolic functions in the human body. The identification of *SOX9^+^* Epi4 cells highlights a key cell population involved in bile acid and hormone metabolism within the biliary epithelial cells, offering enhanced insight into the composition and functional impairment of biliary epithelial cells in NAS patients. By analyzing the cell‒cell communication among epithelial cell subclusters in the normal, STT, and LTT groups, we found that the VEGF, IGF, and HGF signalling pathways were restored after ET. The restoration of these growth factor‐related pathways may promote the regeneration and repair of epithelial cells, thereby facilitating the restoration of normal biliary function.^37^, ^38^

Macrophages are essential in LT because they clear necrotic tissue, regulate inflammation, promote graft repair and regeneration, and participate in immune regulation and the antiviral immune response.^22^ We analyzed the impact of macrophages on NAS and divided macrophages into seven subclusters. Macro2 was more prevalent in the STT group than in the other groups and exhibited antibody presentation characteristics. Macro5, which exhibited insulin receptor recycling effects, and Macro7, which regulated lipid metabolism, were found to be enriched in the LTT group. Lipid metabolism controls the immunosuppressive phenotype of macrophages, while upregulation of insulin regulation by macrophages can effectively alleviate cell steatosis and inflammatory reactions.^39^, ^40^ Several studies have indeed shown that LT recipients have a higher incidence of metabolic syndrome, insulin resistance, and dyslipidemia than the general population.^41^ The increased proportion of Macro5 and Macro7 in the LTT group suggests that long‐term ET may promote macrophage differentiation toward these types, which can improve the inflammatory, immune, and metabolic environment of the bile ducts. Similar to previous studies, Monocle2 analysis revealed a differentiation trajectory of Macro1 and Macro3 subclusters toward Macro5 and Macro7 subclusters, indicating the transition of macrophages from an inflammatory and immune phenotype to a metabolic phenotype.^42^

In NAS patients, there is a significant increase in the number of plasma cells in the bile duct, which leads to robust activation of humoral immunity. Additionally, plasma cells exhibit upregulation of many ERAD and UPR pathways. The UPR serves as an adaptive cellular response for ERAD, reducing the number of misfolded proteins.^43^ However, if the ERAD activity is too great for compensation, the apoptotic pathway is activated, leading to apoptosis. The UPR is crucial for many liver and biliary diseases.^44^ In NAS patients, the infiltration of many plasma cells disrupts the balance between the overall ERAD and UPR, upregulating the apoptotic pathway. Information on the regulation of immune and ERAD reactions by plasma cells provides a valuable reference for the treatment of NAS patients. In addition, we found that as the B‐cell subclusters differentiated into plasma cell subclusters, the ERAD pathway was continuously upregulated. Among the clusters, the *IGHG1^+^ IGHG2^+^* plasma cell cluster exhibited the highest ERAD pathway activation at the differentiation endpoint. After ET, the proportion of *IGHG1^+^ IGHG2^+^* plasma cells was reduced, indicating that inhibiting differentiation in this direction could be attempted as a treatment for NAS.

In summary, we constructed single‐cell atlases of NAS and ET for NAS patients after LT. These atlases provide a rich resource for studying the evolution of cellular compositions, transcriptional networks, and communication patterns during the NAS and ET processes. We have elucidated the mechanisms underlying the evolution of intercellular and intracellular processes caused by NAS and ET. Our findings provide excellent guidance to aid in the discovery of molecular targets and pathways. This study thus provides novel insights and directions for understanding, preventing, and treating NAS.

However, there were a few limitations to our study. It would have been more appropriate to use bile duct samples from non‐NAS liver transplant patients as the normal control group. However, due to ethical considerations, obtaining bile duct samples from non‐NAS liver transplant patients was challenging. Therefore, we used bile duct samples from the donor's bile duct as the normal control group. Additionally, the sample size in this study was not sufficiently large, which may lead to bias in the results due to differences among individuals.

## AUTHOR CONTRIBUTIONS

Zhaoyi Wu, Danqing Liu, Yanjiao Ou, Xiaojun Wang, Zhiyu Chen, Leida Zhang, and Chengcheng Zhang conceptualized and supervised the whole project. Zhaoyi Wu, Zhiyu Chen, Leida Zhang, Xiaojun Wang, and Chengcheng Zhang wrote and revised the manuscript. Zhaoyi Wu and Gang Heng performed analysis of scRNA‐seq. Danqing Liu, Yanjiao Ou, and Leida Zhang obtained the clinical samples. Zeliang Xu, Gang Heng, and Di Jiang collected and analyzed patient data. Zhaoyi Wu, Nengsheng Fu, and Jingyi Wang conducted basic experiments including cell isolation, staining, and RT‐qPCR.

## CONFLICT OF INTEREST STATEMENT

The authors declare no conflict of interest.

## ETHICS STATEMENT

Ethical approval was obtained from Southwest Hospital, Third Military Medical University, Chongqing, China (KY2023016). An informed consent form was signed by every participant.

## Supporting information

Supporting Information

Supporting Information

Supporting Information

Supporting Information

Supporting Information

Supporting Information

Supporting Information

Supporting Information

Supporting Information

Supporting Information

Supporting Information

## Data Availability

The scRNA‐seq data generated in this study have been deposited in the GSA database under accession code HRA005693. And the raw data will be available prior to publication.
