# Supplementary material for: Mechanism and endoscopic‐treatment‐induced evolution of biliary non‐anastomotic stricture after liver transplantation revealed by single‐cell RNA sequencing
Source: Clin Transl Med. 2024 Mar 14;14(3):e1622. doi: 10.1002/ctm2.1622 (PMC10938070; doi:10.1002/ctm2.1622)
Supplement: Supplementary file 11 — Supporting Information [file CTM2-14-e1622-s010.docx]

**Mechanism and endoscopic-treatment-induced evolution of biliary non-anastomotic stricture after liver transplantation revealed by single-cell RNA sequencing**

Zhaoyi Wu^1‡^, Danqing Liu^1‡^, Yanjiao Ou^1‡^, Zeliang Xu^1‡^, Gang Heng^2‡^, Wei Liu^1^, Nengsheng Fu^1^, Jingyi Wang^1^, Di Jiang^1^, Lang Gan^1^, Jiahong Dong^3^, Xiaojun Wang^1^, Zhiyu Chen^1^, Leida Zhang^1^, Chengcheng Zhang^1^

**Table of contents**

Materials and methods......................................................................................2

Supplementary figures......................................................................................7

Supplementary figure legends........................................................................14

Supplementary tables.....................................................................................18

Supplementary references..............................................................................19

Materials and methods

**Immunosuppressive regimen**

Tacrolimus along with mycophenolate mofetil (MMF）was the basic protocol for postoperative immunosuppression. A minority of patients transferred tacrolimus to sirolimus for special reasons including tumor or renal dysfunction. Basiliximab was applied in most patients while steroid was rarely used. All patients received a single intravenous injection of 500-mg methylprednisolone during transplantation. The dosing regimen for basiliximab is to intravenously inject 20mg 2 hours before and on the fourth day after liver transplantation surgery.

**Single cell collection and cDNA amplification**

Bile duct samples were rinsed twice with Dulbecco's modified Eagle’s medium (DMEM, Gibco, 11965092) and cut into 1 mm x 1 mm pieces. Next, 5 mL of a mixed digestion enzyme solution (0.03% collagenase type I, 0.05% collagenase type IV, 0.05% dispase, 0.01% DNase I) was added, and the tissues were incubated at 37°C for 50–70 minutes. The reaction was stopped with DMEM containing 10% fetal bovine serum (FBS, Gibco, A3160802) and the sample was filtered through a 40 µm nylon mesh filter. The precipitate was centrifuged at 350 x g for 5 minutes, and washed twice with DMEM containing 10% FBS. The cells were lysed with red Puregene RBC Lysis Solution (Qiagen, 158106) for 5 minutes at room temperature, and then dead cells and debris were removed with a Miltenyi Biotec Dead Cell Removal Kit (Miltenyi Biotec, 130–090–101). The live cell rate was assessed using trypan blue staining, with a minimum requirement of 85%. Samples of bile ducts from patients firstly receiving ET consist of massive necrotic cells, which is not sufficient for sequencing.

**ScRNA–seq data processing**

We generated scRNA-seq profiles using 10x [Genomics sequencing](https://www.sciencedirect.com/topics/medicine-and-dentistry/genome-sequencing). The raw gene expression matrices from each sample were combined using CellRanger (version 6.1.2), and subsequent analysis was performed in R (version 4.2.3) using Seurat (version 4.3.0) to analyze the gene-barcode matrices. SoupX (version 1.6.2) was used to estimate and eliminate cell–free mRNA contamination in droplet-based single-cell RNA-seq data. All samples were merged into one Seurat object and filtered based on specific criteria (nFeature_RNA > 400, nFeature_RNA < 6000, percent of mitochondrial genes < 0.25 and hemoglobin genes < 0.03). Doublets were identified and removed using DoubletFinder (version 2.0.3). A total of 77,038 cells were retained for further analysis.

**Clustering and dimension reduction**

We employed Seurat's "FindVariableFeatures" function to identify the 2000 most variable genes and performed principal component analysis (PCA), cell clustering, and uniform manifold approximation and projection (UMAP) visualization to determine cell types. Harmony (version 0.1.1) was utilized to mitigate batch effects. The functions FindNeighbors in the Seurat package, with dimensions set to 1:40. And FindClusters, with a resolution set to 0.9, resulted in a total of 25 cell clusters. These clusters were accurately annotated into 16 major cell types using established marker genes. Subsequently, we characterized distinct cell types and subtypes within the samples (**Table S1**).

**Differentially expressed genes (DEGs) analysis**

We utilized Seurat's "FindMarkers" function to detect DEGs between the NAS and normal groups and the STT and LTT groups. A LogFC value and an adjusted *P*-value for each DEG were calculated by the Wilcoxon rank–sum test. Genes with |avg_logFC| > 0.25 and p_val_adj < 0.01 were selected.

**Gene function analysis**

We utilized ClusterProfiler (version 4.6.2) to perform enrichment analysis, which helped us identify enriched biological processes and pathways associated with the different cell types and DEGs ^1^. Cytoscape (version 3.10.0) was used to create visual representations of complex networks and biological pathways ^2^.

**Cell-cell interaction analysis**

CellChat (version 1.6.1) is a publicly available database that contains information on receptors, ligands, and their interactions, including subunit structures that accurately represent heteromeric complexes ^3^. We utilized CellChat to perform cell**‒**cell interaction analysis, which enabled us to gain insights into the potential communication and signaling pathways between different cell types.

**Analysis of single-cell trajectories**

Monocle (version 2.24.0) was used for differentiation trajectory and pseudotime analysis of macrophages, B cells and plasma cells ^4^.

**Gene set variation analysis (GSVA)**

We used GSVA to comprehensively assess the expression levels of individual genes in pathways and assign pathway activity estimates for individual cells. We performed GSVA using GSVA software (version 1.46.0) and downloaded the screened signaling pathway gene sets from the Molecular Signatures Database (MSigDB) ^5^. This approach allowed us to identify any significant differences in pathway activity between different cell types.

**Assessment of the ratio of observed to expected cell number (Ro/e) for each cluster**

To determine the tissue preference of each cell cluster, we calculated the Ro/e and considered a cluster enriched if the Ro/e ratio was > 1 ^6^. Heatmaps were generated using pheatmap (version 1.0.12).

**Hematoxylin and eosin (H&E) staining**
The sample is fixed using formalin to preserve its structure. It is then dehydrated through a series of concentration gradient ethanol washes (75%, 85%, 90%, 100%) to remove water. The sample is embedded in paraffin to provide support for sectioning. Thin sections of the embedded sample are cut using a microtome and placed on glass slides. The sections are stained with hematoxylin and eosin to visualize structures such as cell nuclei and cytoplasm. The stained sections are then dehydrated and cleared again using xylene and different concentration gradient ethanol to prepare for mounting. The prepared slides are analyzed and interpreted under a microscope.

**Immunofluorescence (IF)**

Frozen sections were washed in PBS and fixed in 4% paraformaldehyde in PBS, and paraffin–embedded sections were deparaffinized and rehydrated. Antigen retrieval was performed, and the sections were permeabilized and blocked. The sections were incubated with primary antibodies overnight and then with secondary antibodies. The relevant antibodies and concentrations can be found in Table S2.

**Immunohistochemistry (IHC)**

For immunohistochemistry, paraffin sections were subjected to incubation with primary antibodies at 4°C overnight, followed by binding with biotinylated secondary antibodies and DAB color development.

**Reverse transcription polymerase chain reaction (RT-qPCR)**

Total RNA was extracted from tissue using an RNAiso Plus kit (TaKaRa, Code No. 9019) and reverse–transcribed into cDNA using a PrimeScript RT Reagent Kit with gDNA Eraser (TaKaRa, Code No. RR047A). Quantitative PCR was performed using a TB Green Premix Ex Taq II kit (TaKaRa, Code No. RR820A) on a CFX96 Real–Time PCR Detection System following the manufacturer's instructions. Each sample was tested in triplicate. The relevant human and rat primers can be found in Table S3.

**Western blotting**

Tissues underwent lysis using RIPA lysis buffer (Beyotime, Shanghai, China) supplemented with a protease inhibitor mix (Beyotime, Shanghai, China) for 30 minutes at 4 °C, followed by centrifugation at 13,000 g for 15 minutes at 4 °C. Protein concentrations were determined utilizing a BCA Protein Assay Kit (Beyotime, Shanghai, China). The proteins were subsequently denatured, separated by SDS-PAGE (Beyotime, Shanghai, China), and transferred onto NC membranes (GE Healthcare, UK). Following blocking with 5% skim milk, NC membranes were subjected to overnight incubation at 4 °C with specified primary antibodies. Subsequently, membranes were exposed to suitable secondary antibodies and scanned using a ChemiDoc imaging system (Bio-Rad, Hercules, CA, USA).

**Flow cytometry analysis**

Tissue digestion and cell collection were conducted using the identical procedures employed in single-cell RNA sequencing. Cells were stained for 20 min at room temperature, with extracellular panel including antibodies specific for: CD45 PerCP-Cy5.5, CD3 BV421, CD4 AF647, CD8 PerCP, CD-11b APC, Ly6c FITC, CD19 AF488 and CK7 PE. Flow cytometry acquisition was performed in a FACSverse flow cytometer (BD Biosciences) and data were analyzed using FlowJo software (v10.7.2).

Supplementary figures
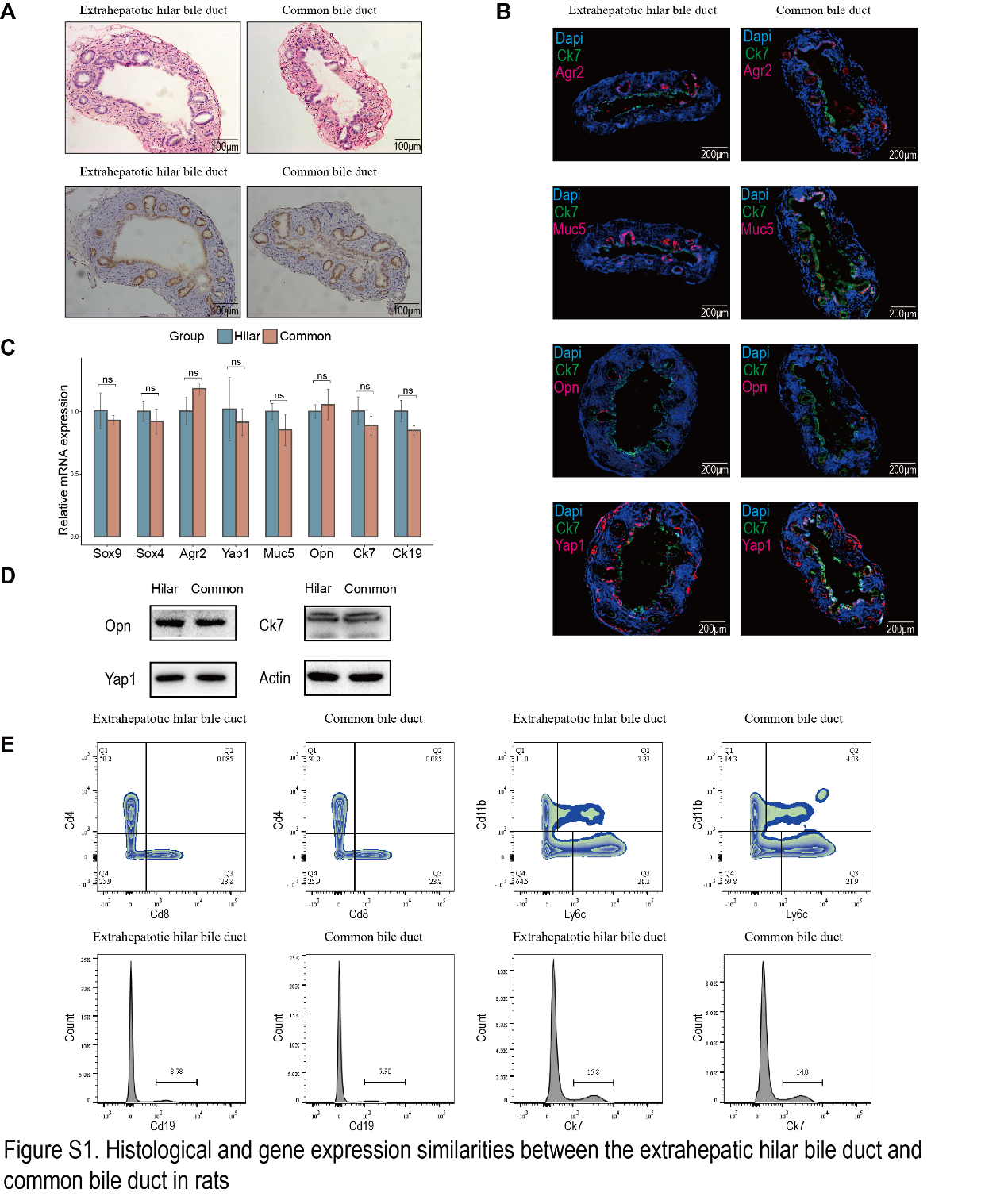

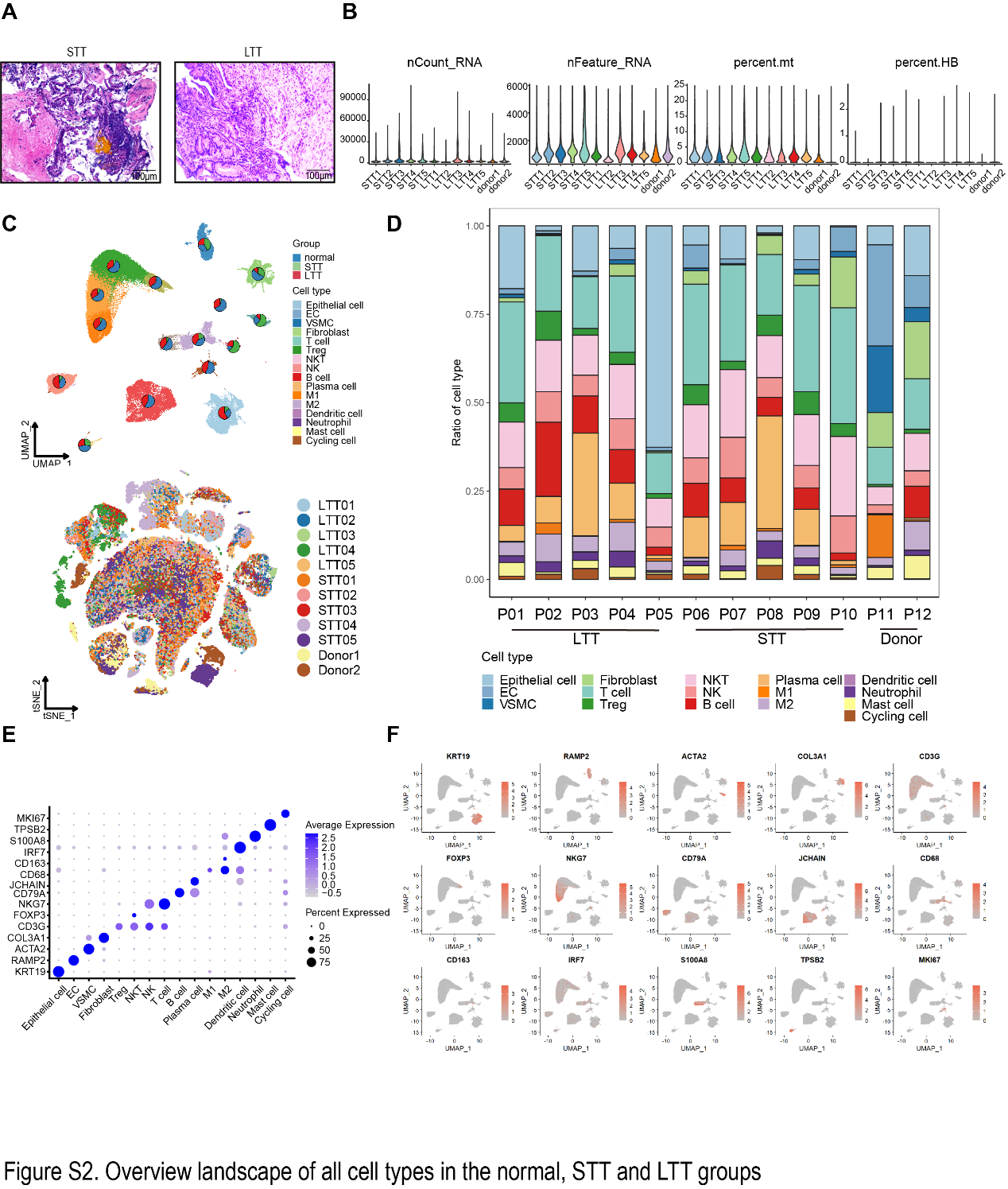

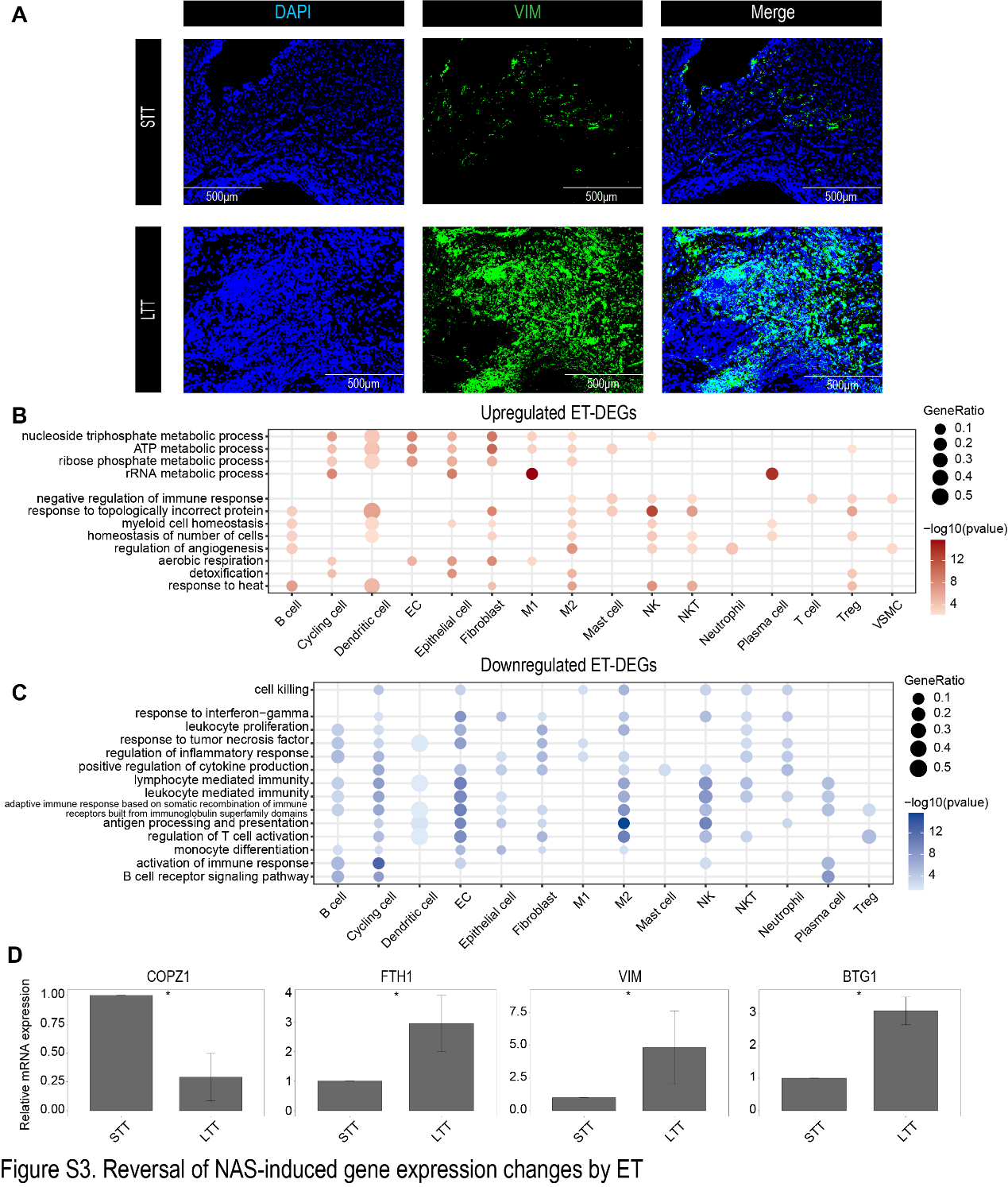

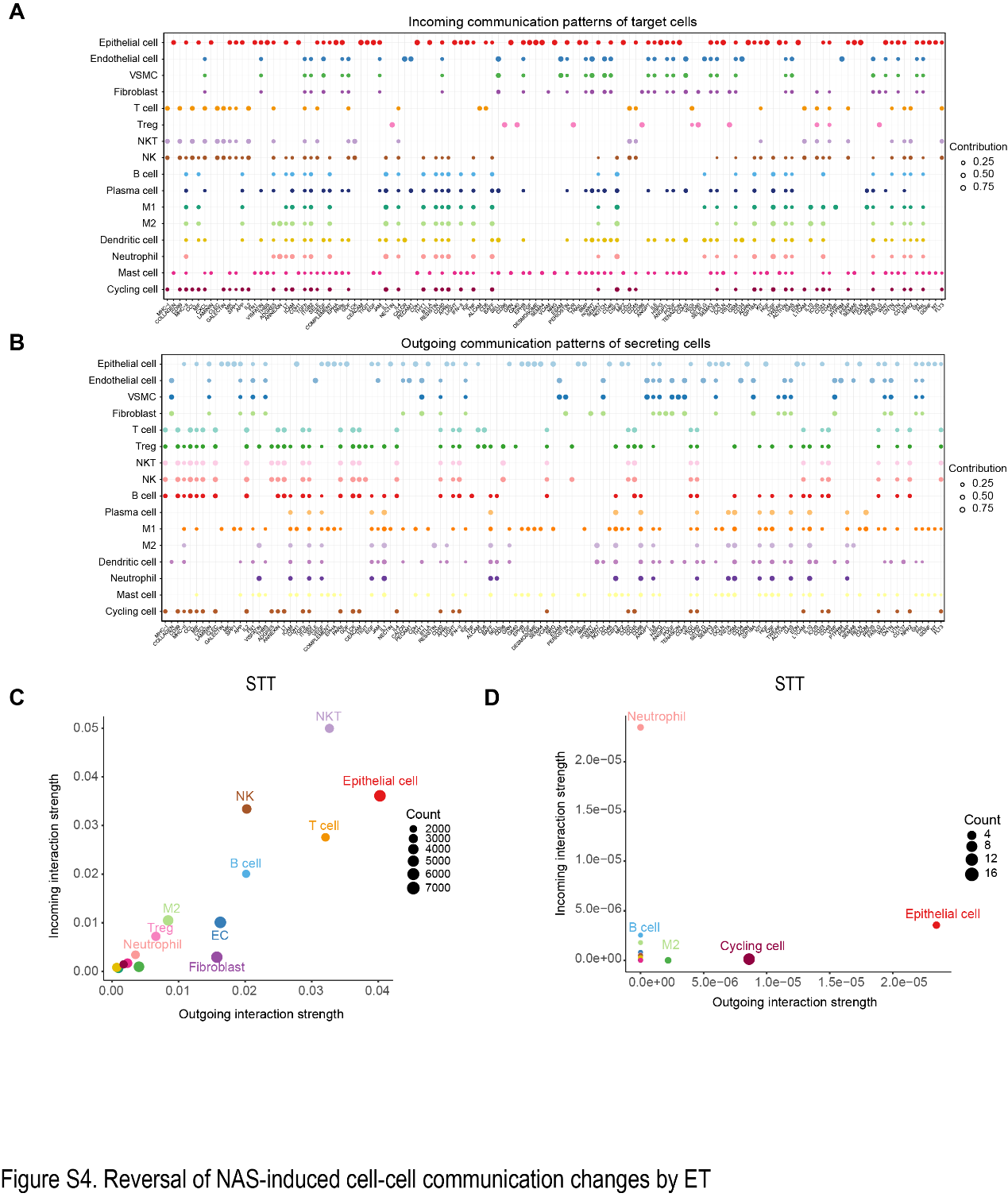

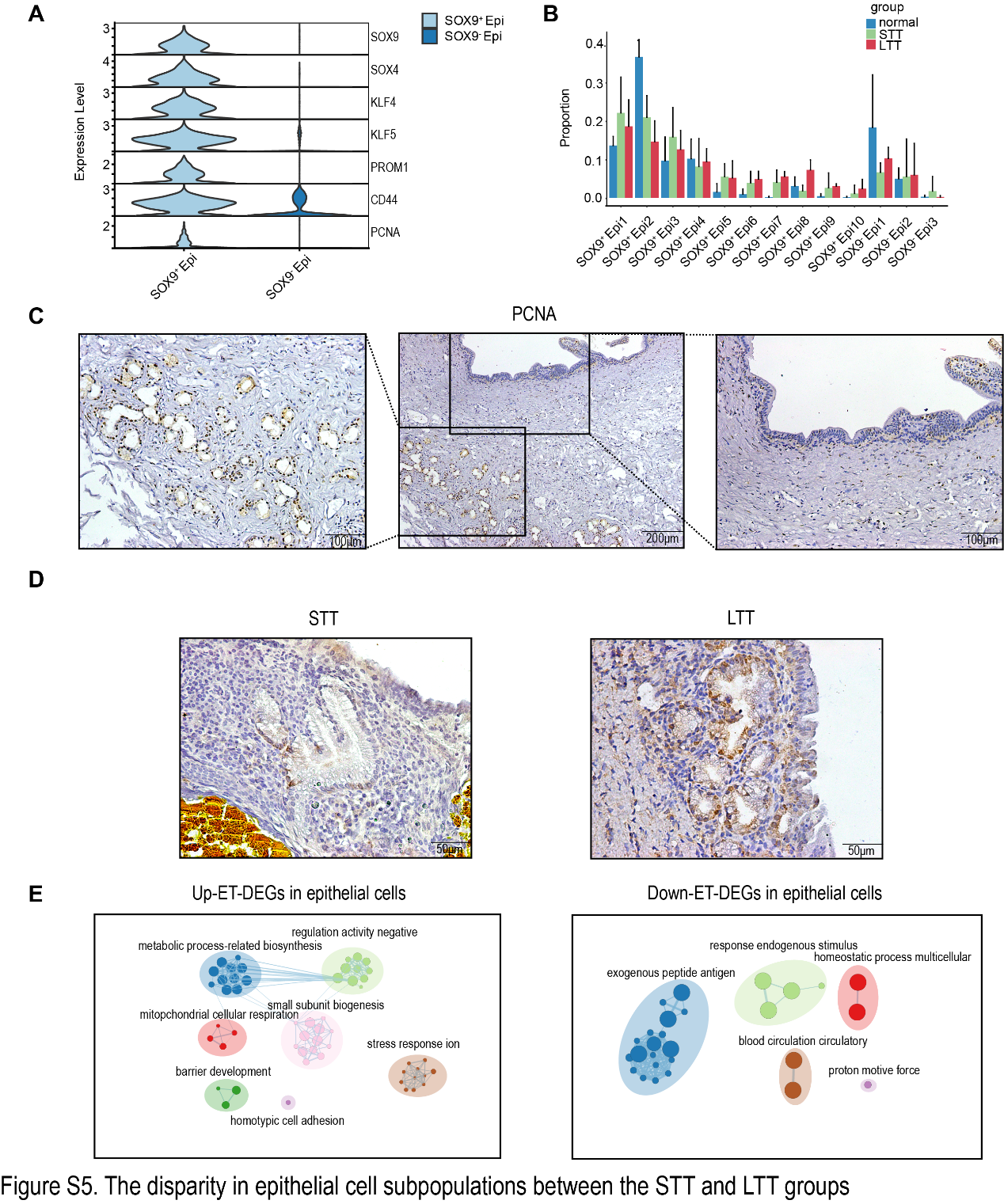

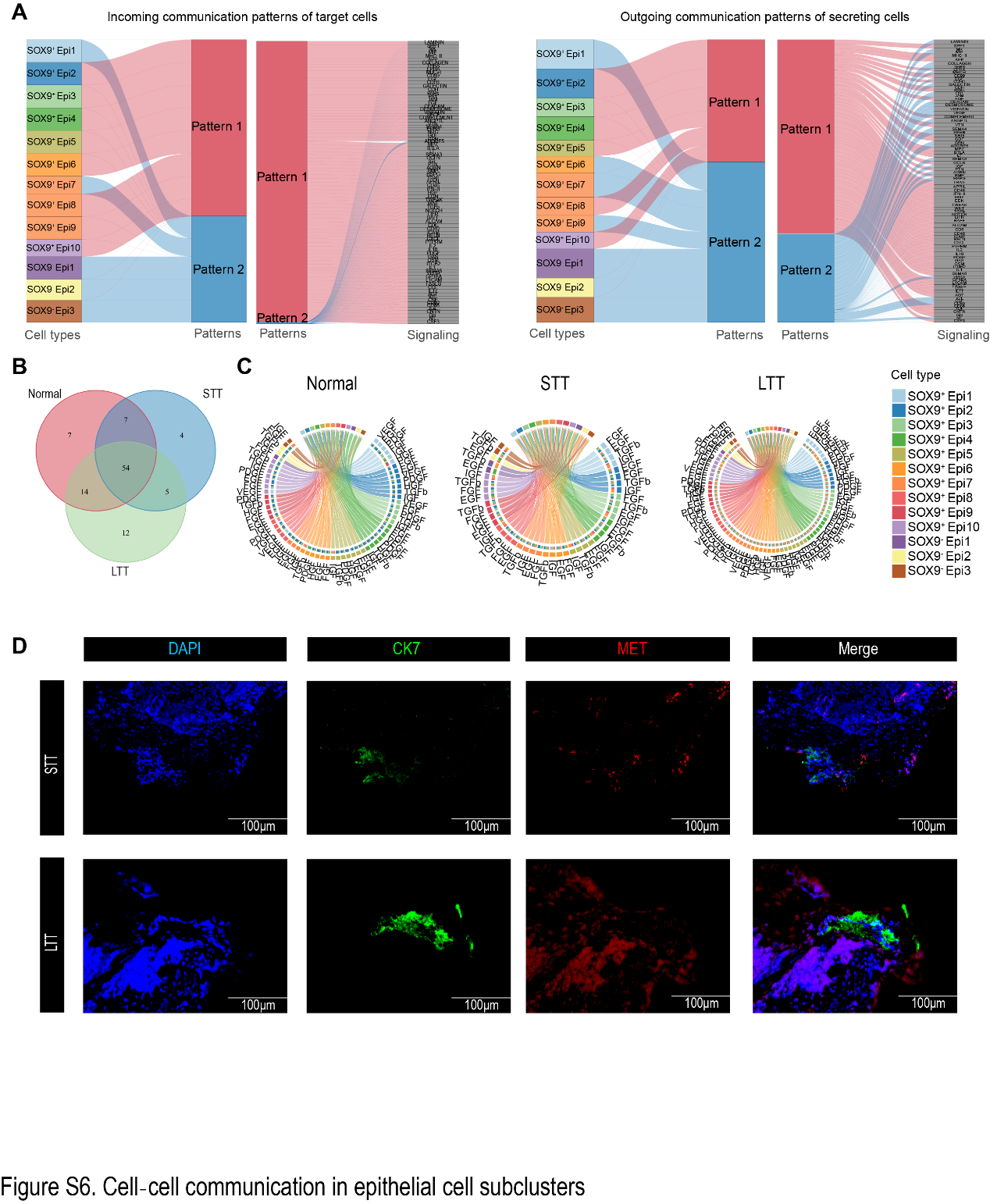

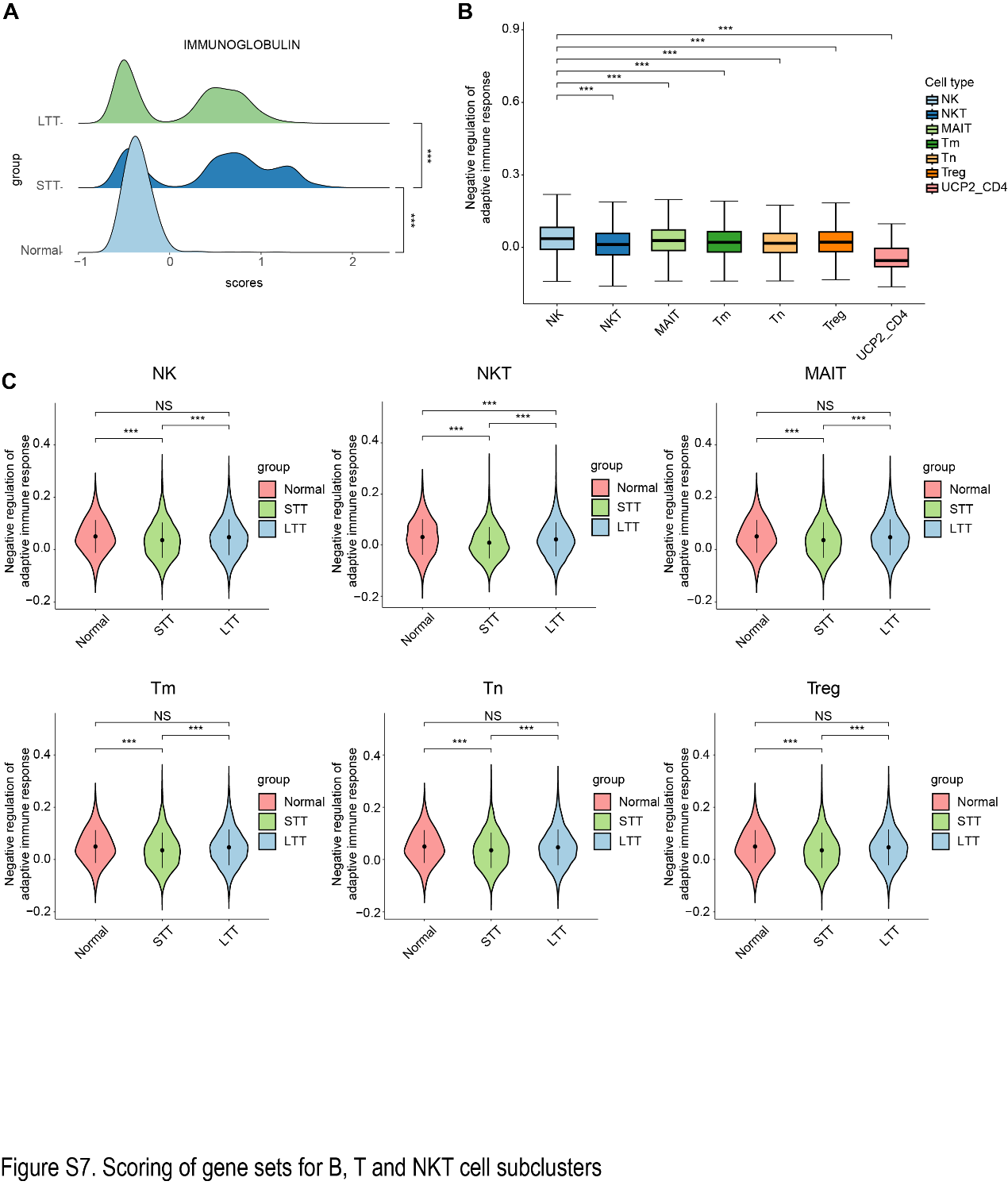


Supplementary figure legends

**Figure S1. Histological and gene expression similarities between the extrahepatic hilar bile duct and common bile duct in rats**

(A) Histological images of the extrahepatic hilar bile duct and common bile duct in rats (above). Two groups of large duct lumens are composed of a single layer of tall columnar epithelium, and the epithelium underneath contains abundant peribiliary glands. IHC staining showing the expression of *Sox9* in the extrahepatic hilar bile duct and common bile duct in rats (below). Two groups show that the peribiliary gland cells express higher levels of *Sox9* compared to mature epithelial cells. Scale bars, 100 μm.

(B) IF staining showing the expression of *Agr2, Muc5, Opn and Yap1* in the extrahepatic hilar bile duct and common bile duct. *Agr2* and *Muc5* are both more expressed in the peribiliary glands. And *Yap1* is expressed in both peribiliary glands and lumens. Scale bars, 200 μm.

(C) Bar plots showing the *Sox9, Sox4, Agr2, Yap1, Muc5, Opn, Ck7 and Ck19* genes relative mRNA expression levels detected by RT-qPCR between the extrahepatic hilar bile duct and common bile duct. There are no significant differences in the developmental markers (*Sox9, Sox4, Agr2, Yap1, Muc5, Opn*) of the bile duct between the two groups. NS, no significance; *P < 0.05; **P < 0.01; ***P < 0.001.

(D) Western blotting showing the protein levels of *Opn, Yap1* and *Ck7* in the extrahepatic hilar bile duct and common bile duct.

(E) Flow cytometry showing the proportions of important immune cells (Cd4^+^ T, Cd8^+^ T, Cd11b^+^ monocyte, Ly6c^+^ macrophage, B cell) and epithelial cells in the extrahepatic hilar bile duct and common bile duct.

**Figure S2. Overview landscape of all cell types in the normal, STT and LTT groups**

(A) Histological images of the peribiliary glands of the STT and LTT groups. The morphology of the peribiliary glands is disrupted, with shedding cells and a large amount of inflammatory cell infiltration in the lumen (STT), and the morphology of the peribiliary glands is normal, and the peribiliary gland cells maintain a single layer of columnar epithelium (LTT). Scale bars, 100 μm.

(B) Violin plots showing the quality control features, including the number of UMI counts, gene features, percentage of mitochondrial genes and percentage of hemoglobin genes of each patient.

(C) UMAP plots of 77,038 high-quality single cells, and pie plots showing the proportion of each cell type within each group (above). tSNE plots of 77,038 cells, annotated and colored by patient origin (below).

(D) Bar plots showing the proportions of cell types in each patient.

(E) Dot plots showing the marker genes of each cell type.

(F) UMAP plots of the expression levels of marker genes, defined for all cell

types.

**Figure S3. Reversal of NAS-induced gene expression changes by ET**

(A) IF staining showing the expression of VIM in bile duct tissue of the STT and LTT groups. Scale bars, 500 μm.

(B) Dot plots summarizing common biological pathways enriched among upregulated ET-DEGs in major cell types.

(C) Dot plots summarizing common biological pathways enriched among downregulated ET-DEGs in major cell types.

(D) Bar plots showing the *COPZ1*, *FTH1*, *VIM* and *BTG1* genes relative mRNA expression levels detected by RT-qPCR between STT group and LTT group. NS, no significance; *P < 0.05; **P < 0.01; ***P < 0.001.

**Figure S4. Reversal of NAS-induced cell-cell communication changes by ET**

(A) Dot plots summarizing the overall incoming communication of target cells.

(B) Dot plots summarizing the overall outgoing communication of secreting cells.

(C) Scatterplot showing the contribution of each cell type to all cell**-**cell communication mediated communication in the STT group.

(D) Scatterplot showing the contribution of each cell type to VWF, PRL, and SECTIN mediated communication in the STT group.

**Figure S5. Disparity in epithelial cell subpopulations between the STT and LTT groups**

(A) Violin plots showing the expression of *SOX9*, *SOX4*, *KLF4*, *KLF5*, *PROM1*, *CD44* and *PCNA* in *SOX9^+^* and *SOX9^-^* epithelial cells.

(B) Bar plots showing the proportions of epithelial cell subclusters in the three groups.

(C) IHC staining showing the expression of *PCNA* in bile duct tissue of donors. Scale bars, 200 and 100 μm.

(D) IHC staining showing the expression of *SOX9* in bile duct tissue of the STT and LTT groups. Scale bars, 50 μm.

(E) Network graph illustrating representative GO terms and pathways of upregulated (left) and downregulated (right) ET-DEGs in epithelial cells. This plot created with Cytoscape.

**Figure S6. Cell‒cell communication in epithelial cell subclusters**

(A) River plot summarizing the overall incoming (left) and outgoing (right) communication of target and secreting cells in the LTT group.

(B) Venn diagram showing the differences in cell**‒**cell communication of epithelial cell subclusters among three groups.

(C) Chord plots showing cell**‒**cell communication mediated by the VEGF, PDGF, HGF, TGF-β, FGF, IGF and EGF pathways across all epithelial cell subclusters in three groups.

(D) IF staining for *MET*, *CK7* and *DAPI* in bile duct tissue of the STT and LTT groups. Scale bars, 100 μm.

**Figure S7. Scoring of gene sets for B, T and NKT cell subclusters**

(A) Ridge plots showing the immunoglobulin-related gene scores of B cells among the three groups. NS, no significance; *P < 0.05; **P < 0.01; ***P < 0.001.

(B) Boxplots showing negative regulation of adaptive immune response scores for T and NK subclusters, by comparing NK cells with other T and NK subclusters. NS, no significance; *P < 0.05; **P < 0.01; ***P < 0.001.

(C) Violin plot comparing negative regulation of adaptive immune response scores for all T and NK subclusters among the three groups. NS, no significance; *P < 0.05; **P < 0.01; ***P < 0.001.

Supplementary tables

**Table S1.** Markers of cell type defined by scRNA-seq data in bile duct.

**Table S2.** Antibodies used for IF, IHC, WB and flow cytometry.

**Table S3.** Human primers used for RT-qPCR.

**Table S4.** Comparisons of 35 NAS patients’ clinical characteristics between the STT and LTT groups.

**Table S5.** Comparisons of sequenced patients’ clinical characteristics between the STT and LTT groups.

**Table S6.** Clinical characteristics of two donors.

**Table S7.** Quality of cells obtained from sample dissociation.

**Table S8.** NAS-DEGs from 16 distinct cell types.

**Table S9.** ET-DEGs from 16 distinct cell types.

**Table S10.** Cell-cell communication among the normal, STT and LTT groups.

The details are presented in the EXCEL files.

Supplementary references

1. Wu T, Hu E, Xu S, et al. clusterProfiler 4.0: A universal enrichment tool for interpreting omics data. *Innovation (Camb).* 2021;2(3):100141.

2. Shannon P, Markiel A, Ozier O, et al. Cytoscape: a software environment for integrated models of biomolecular interaction networks. *Genome Res.* 2003;13(11):2498-2504.

3. Jin S, Guerrero-Juarez CF, Zhang L, et al. Inference and analysis of cell-cell communication using CellChat. *Nat Commun.* 2021;12(1):1088.

4. Qiu X, Mao Q, Tang Y, et al. Reversed graph embedding resolves complex single-cell trajectories. *Nat Methods.* 2017;14(10):979-982.

5. Subramanian A, Tamayo P, Mootha VK, et al. Gene set enrichment analysis: a knowledge-based approach for interpreting genome-wide expression profiles. *Proc Natl Acad Sci U S A.* 2005;102(43):15545-15550.

6. Guo X, Zhang Y, Zheng L, et al. Global characterization of T cells in non-small-cell lung cancer by single-cell sequencing. *Nat Med.* 2018;24(7):978-985.
